# Calcium Handling Machinery and Sarcomere Assembly are Impaired Through Multipronged Mechanisms in Cancer Cytokine‐Induced Cachexia

**DOI:** 10.1002/jcsm.13776

**Published:** 2025-04-04

**Authors:** Luis Vincens Gand, Chiara Lanzuolo, Mugeng Li, Valentina Rosti, Natalie Weber, Dongchao Lu, Christian Bär, Thomas Thum, Andreas Pich, Theresia Kraft, Mamta Amrute‐Nayak, Arnab Nayak

**Affiliations:** ^1^ Institute of Molecular and Cell Physiology Hannover Medical School Hannover Germany; ^2^ Istituto Nazionale Genetica Molecolare ‘Romeo ed Enrica Invernizzi’ Milan Italy; ^3^ Institute of Biomedical Technologies National Research Council Milan Italy; ^4^ Institute of Molecular and Translational Therapeutic Strategies Hannover Medical School Hannover Germany; ^5^ Fraunhofer Institute for Toxicology and Experimental Medicine (ITEM) Fraunhofer Cluster of Excellence Immune‐Mediated Diseases (CIMD) Hannover Germany; ^6^ Institute of Toxicology, Core Facility Proteomics Hannover Medical School Germany

**Keywords:** calcium homeostasis, cancer secreted pro‐inflammatory cytokine‐induced cachexia, chromatin organization, proteostasis, RNA polymerase II, sarcoplasmic reticulum

## Abstract

**Background:**

Cachexia is a severe form of muscle wasting disorder particularly observed in patients with advanced cancer. The absence of effective strategies to ameliorate cachexia indicates our poor understanding of the mechanisms of cachexia. By employing system‐wide approaches, we investigated molecular mechanisms underlying cancer secreted pro‐inflammatory cytokine‐induced cachexia (CIC).

**Methods:**

As cellular model systems, we employed mouse satellite stem cell‐derived primary muscle cells, mouse C2C12 myoblast progenitor cell‐derived myotubes, and neonatal rat cardiomyocytes. We induced CIC by incubating striated muscle cells with pro‐inflammatory cytokines TNF‐α and IFN‐γ. To understand the physiological effects of CIC, we probed the contractile properties of muscle cells following electrical stimulation and measured intracellular calcium transients. Effects of CIC on sarcomere organization were monitored by confocal microscopy. Large‐scale quantitative proteomics and RNA sequencing assays enabled us to examine molecular mechanisms underlying CIC. Using chromatin immunoprecipitation experiments, chromatin signalling and modulation of epigenetic marks on muscle‐specific genes were investigated.

**Results:**

Here, we observed a drastic loss of striated muscle cell contraction in CIC, primarily, due to acutely disorganized sarcomere structures and impeded calcium handling process. In calcium transients, the extent of calcium (Ca^2+^) release, as indicated by the calcium amplitude during the excitation–contraction coupling (ECC) process, was reduced (19.6 ± 2.35% in control to 8.6 ± 1.52% in CIC, *p* = 4.8 * 10^−11^). Kinetics of calcium transients, i.e., the Ca^2+^ release rate (26 ± 0.5 ms in control to 29 ± 5.1 ms in CIC, median *p* = 0.014), and calcium re‐uptake rate (137 ± 13 ms in control to 185 ± 24 ms in CIC, *p* = 0.032) were both prolonged. Proteomic analysis showed altered proteostasis in CIC, particularly related to sarcomere and sarcoplasmic reticulum (SR). Transcriptomic analysis unravelled upstream deregulation of global transcriptional events for sarcomeric and SR genes. Mechanistically, chromatin loading of transcriptionally active RNA Polymerase II on muscle‐specific genes, including *Myh1* and *Atp2a1*, was impeded. This was due to diminished transcriptionally active epigenetic marks H3K4 trimethylation on *Myh1* and *Atp2a1*, resulted in lower transcriptional activity of these muscle‐specific genes in CIC and ultimately reduced MyHC‐IId molecular motor protein and SERCA1 protein levels.

**Conclusions:**

Our top‐down approach elucidated that the altered transcriptional mechanism and proteomic state perturbed functionally related machinery responsible for calcium handling and sarcomere organization in CIC. Knowledge of the underlying cause of muscle mass loss and compromised muscle function is key for developing therapeutic solutions to ameliorate cachectic conditions.

## 1 Introduction

The precisely arranged sarcomeres are the fundamental units of striated muscle cells that produce force from ATP‐dependent cross‐bridge cycling between actin (thin filaments) and myosin (thick filaments) to bear load and drive movement. During excitation–contraction coupling (ECC) process, calcium (Ca^2+^) is released from the sarcoplasmic reticulum (SR) through the ryanodine receptor (RyR1), activating thin filament and enabling acto‐myosin cross‐bridge cycling, and hence myocyte contraction [1, 2]. The dihydropyridine receptor (DHPR) α1s subunit physically interacts with RyR1, inducing an opening of the channel as the result of an action potential [1]. Muscle relaxation follows the transfer of Ca^2+^ ions to the SR through the calcium ATPase pump SERCA1. Thus, a precise sarcomeric assembly, a proper Ca^2+^ transient, and correct interplay between these two systems is critical for primary muscle cell functions, i.e., contraction and force generation. Molecular insights in various aspects of these processes remain poorly determined. A detailed understanding of it is critical to apprehend not only muscle physiology but muscle wasting conditions as well.

Cancer triggers cachexia, which is a severe muscle wasting disorder associated with up to 80% of cancer patients [3]. Cancer cytokine‐induced cachexia (CIC) is defined by ongoing involuntary loss of muscle and/or fat mass and is not reversible by common treatments [4], with an estimated high mortality rate ranges from 20% to 50% in cancer patients depending on cancer types [5]. The effective dose of cancer therapeutics, particularly chemotherapy, is calculated based on body surface area [6, 7]. Thus, CIC additionally aggravates the patient's responsiveness to therapies [8]. Tumour‐released pro‐inflammatory cytokines, such as tumour necrosis factor alpha (TNF‐α), interferon gamma (INF‐γ), interleukin 6 (IL‐6), induce degradation of myofibril proteins, especially myosin heavy chain (MyHC), through NF‐κB (nuclear factor ‘kappa‐light‐chain‐enhancer’ of activated B‐cells)‐MuRF1 (muscle RING‐finger protein‐1) ubiquitin‐proteasome pathway [9]. Furthermore, NF‐κB downregulates the master transcription factor MyoD and thereby suppresses skeletal muscle differentiation of myogenic progenitor cells [10, 11]. Besides Murf1, other E3 ligases including Atrogin1 and UBR2 are also upregulated and modify thick filament proteins for degradation [12, 13]. Pro‐inflammatory cytokines also contribute to insulin resistance and suppression of the insulin‐like growth factor I (IGF1)‐Akt pathway, further deteriorating the catabolic condition [14]. Moreover, upregulation of the metal‐ion transporter ZRT‐ and IRT‐like protein 14 (ZIP14) has been shown in cachectic skeletal muscles of mice and in human patients with metastatic cancer in response to cancer‐induced TNF‐α and TGF‐β cytokine release [15]. Despite all these important findings, no effective treatment regimens to target CIC in human are currently available. It points toward previously undetermined molecular mechanisms that are linked with CIC.

In the present study, we observed a loss of muscle cell function, in both skeletal and cardiac muscle cells, in CIC. At physiological level, we identified a deregulated calcium homeostasis and complete disorganization of sarcomeric structures in CIC. Our system‐wide approach showed that CIC reorients the transcriptional state of distinct major muscle‐specific genes critical for calcium homeostasis and muscle contraction. Furthermore, our investigation unravelled chromatin‐related events of distinct muscle‐specific genes, as the initial trigger of CIC.

## 2 Methods

### 2.1 Cell Culture and Induction of Cachexia

Murine C2C12 myoblast progenitor cells were seeded in T75 flasks at 8 × 10^6^ cells or in six‐well plates at 1 × 10^6^ cells per well in Dulbecco's modified eagle medium (DMEM) containing 4.5 g/L glucose, 15% foetal bovine serum and 1% Penicillin–Streptomycin. Myogenic differentiation was induced by switching to differentiation medium containing 2% horse serum and the medium was changed daily. The cells were differentiated for 5 days to generate mature myotubes. Cachexia was induced by the addition of 10 ng/mL tumour necrosis factor α (TNF‐α; Roche, Switzerland, cat. no. 11271156001) and 100 ng/mL Interferon γ (INF‐γ; Abcam, UK, cat. no. ab9922) for 48 h. For contractility assays, analysis of Ca^2+^ transients and immunofluorescence assays, C2C12 cells were seeded on 10 or 18 mm glass coverslips coated with laminin.

### 2.2 Satellite Stem Cell Isolation, Differentiation and Treatment

Satellite stem cell isolation was performed according to the previous study [16]. After the cells reached ~90% confluency, they were differentiated into myotubes using DMEM supplemented with 5% HS and 1% Pen/Strep for 4 days. Differentiated myotubes were treated for 48 h with 10 ng/mL mTNF‐α and 100 ng/mL IFNγ in fresh DMEM supplemented with 5% HS and 1% penicillin/streptomycin. The addition of fresh cytokines is repeated at 24‐h intervals. Corresponding volume of deionized water was used as vehicle control. Myotubes cultured on 24‐well dishes were collected with trypsin EDTA 0.25% for 3 min at 37°C and resuspended in media containing serum. After centrifugation, the supernatant was discarded and the pellet was frozen at −80°C.

### 2.3 Analysis of Ca^2+^ Transients

Differentiated myotubes on glass coverslips were placed in a custom‐made perfusion chamber set to 37°C with the NBD TC2 Bip temperature controller. The cells were perfused in a solution containing 20 mM HEPES, 1.2 mM NaH_2_PO_4_, 0.66 mM MgSO_4_, 117 mM NaCl, 5.7 mM KCl, 5 mM Na‐Pyruvate, 1.25 mM CaCl_2_, 10 mM Creatin and 10 mM Glucose set to pH 7.4 at 37°C. To induce ryanodine receptor (RyR)‐dependent Ca^2+^ release, the cells were switched to a perfusion solution additionally containing 10 mM caffeine. Prior to measurements, the cells were loaded for 60 min with 5 μM Fura‐2‐AM in serum‐free DMEM in the presence of 2.5 mM Probenecid and 0.025% (w/v) Pluronic F‐127 and washed twice for 15 min with serum‐free DMEM. Ca^2+^ transients were recorded on a MyoCam optical contraction analysis system (IonOptix, USA). The cells were paced with a MyoPacer EP field stimulator at 1 Hz, 25 V, and a pulse duration of 4 ms in TTL mode. Single cell fluorescence measured at 510 nm was recorded after excitation at 340 nm (to detect bound Ca^2+^) and 380 nm (to detect free Ca^2+^). For data analysis the IonWizard software was used. After subtracting the background fluorescence of non‐fura‐2‐treated myotubes the following parameters were calculated: (I) ‘time to peak’ indicates the time between baseline and peak of Ca^2+^ transients, (II) peak height or amplitude compared to baseline in percentage, and (III) time constant (τ) for Ca^2+^ reuptake is estimated by fitting the signal decay with the single exponential decay function. “For the experiments involving Thapsigargin (Tg), fura2 loaded cells were placed in a custom‐made chamber with Ca^2+^‐free extracellular solution at 37°C. Ca^2+^ transient measurements were started immediately after keeping the cell in Ca^2+^‐free extracellular solution without pacing. After 60 s of recordings, 2 μM Tg was added to the Ca^2+^‐free extracellular solution and recorded (without pacing) for a further 120 s.

### 2.4 Mass Spectrometric and Data Analysis

Proteins were mixed and alkylated by acrylamide and further processed by SDS‐PAGE and in gel digested as described [17]. Peptide samples were analysed with a shot‐gun approach and data dependent analysis in an LC–MS system (RSLC, Orbitrap Exploris 240, both Thermo Fisher) as described recently [18]. Raw MS data was processed using Max Quant (version 2.0) [19], and Perseus software (version 2.0.6.0) [20] and mouse entries of uniprot DB. Proteins were stated identified by a false discovery rate of 0.01 on protein and peptide level. After MaxQuant analysis, the proteinGroups (PGs) file was loaded into Perseus. All PGs identified by site, reverse, and potential contaminants were removed. For further analysis, only PGs were considered, which were quantified in all three replicates of at least one condition. Intensities were transformed into log_2_ values, normalized by subtracting the column median, and missing values imputed from normal distribution separately for each column. Expression changes in cachectic condition was calculated by subtracting the control from cachectic intensities for each PG. Log_2_ of 1 was chosen as a cut‐off value for significant enrichment and significance was calculated by two‐way ANOVA with a cut‐off of *p* ≤ 0.05.

### 2.5 Chromatin Immunoprecipitation

Chromatin immunoprecipitation (ChIP) was performed as in Ref. [21] with minor modifications. Briefly, chromatin was sheared in a Covaris M220 focused sonicator for 12 min set to 10% duty factor, 75 W peak incident power, 200 cycles per burst, at 7°C. Sheared chromatin was used for immunoprecipitation assays (using specific antibodies as described in the figures). The DNA from input and immunoprecipitated chromatin was isolated by incubating Protein G dynabeads (that captured immunoprecipitated chromatin‐antibody complex) with 100 μL 10% (w/v) Chelex‐100, for 10 min at 1200 rpm and 95°C in a thermomixer. The beads were spun down and the reversed cross‐linked DNA was used for qPCR. The diluted input was adjusted to 100% by subtracting 5.05 of the raw C_p_ values (Log_2_ (33.3) = 5.05). The percentage of input was calculated by 100 * 2^(C_p_ (adjusted input) – C_p_ (ChIP eluates)).

### 2.6 Statistical Analysis

Most figures were prepared with the tidyverse package [22] for R.

Remaining parts of methods (including isolation of neonatal rat cardiomyocytes, Immunofluorescence assay and confocal imaging, RNAseq and cell pacing assay, western blotting and real time qPCR, immunoprecipitation and siRNA transfection) and detailed sources of reagents are described in the supplemental file.

## 3 Results

### 3.1 CIC Impairs Contractile Function of Skeletal Muscle

**FIGURE 1:**
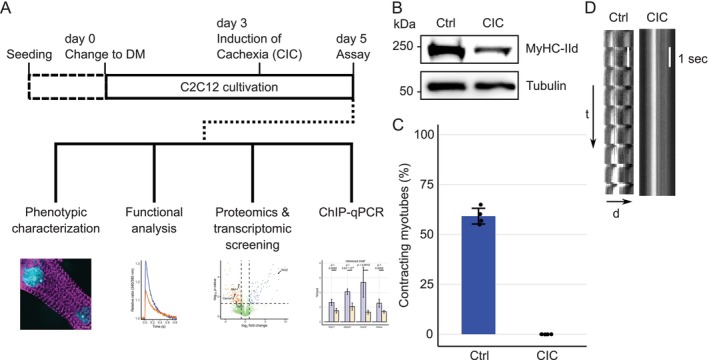
CIC impedes contraction in skeletal myotubes. (A) Scheme illustrating C2C12 progenitor myoblast differentiation in myotubes, induction of CIC and the experimental setup. (B) Immunoblot of cell lysates from myotubes showing reduction of MyHC‐IId in CIC, tubulin is shown as loading control. Representative blot from three biological replicates is shown. (C) Percentage of contracting myotubes when placed under electrical stimulation at 40 V, 1 Hz and 4 ms pulse duration. Control (574) and 521 CIC myotubes from two biological replicates were analysed in total. (D) Representative kymographs showing status of contraction in control vs. CIC cells. The kymographs (time vs. distance plot indicates the movement of intracellular structures of the myotubes in response to the electrical stimulation) were plotted by drawing a line across the myotube/s to check the changes in the intensity along the plotted line over a stack of 100 images or frames. Changed intensity is indicative of a change in the position of respective organelle or structure during the representative contraction event. As seen for control cells, along the vertical axis, the change in the intensities with horizontal shifts (peaks) at regular intervals indicate ‘contraction’. The same horizontal peaks were not visible in CIC, indicating a loss of contraction. Abbreviations: CIC, cancer secreted pro‐inflammatory cytokine‐induced cachexia; Ctrl, control; DM, differentiation medium; MyHC‐IId, myosin heavy chain IId.

To study physiological effects of CIC in skeletal muscle cells, murine C2C12 progenitor myoblasts were differentiated into myotubes. Taking cues from previously established methods mimicking cancer microenvironment [10, 12], we triggered cachexia in differentiated myotubes using pro‐inflammatory cytokines TNF‐α and IFN‐γ for 48 h (Figure 1A). Myotubes with 48 h of cytokine treatment showed a prominent reduction of the molecular motor protein MyHC‐IId protein level (Figure 1B). Loss of MyHC is a hallmark of cachexia. To test the physiological impact of CIC on muscle cell function, myotubes were placed under electric stimulation and paced at 40 V and 1 Hz to induce contraction by mimicking nerve action potential and activating ECC. We observed that about 60% of myotubes in control (Ctrl) conditions exhibited synchronized contraction. Surprisingly, we did not observe any contractile cells in CIC, suggesting that CIC severely impaired the contractile property of skeletal muscle cells (Figure 1C,D and Movie S1). Noteworthy, the dose of cytokines and combination used in this study were designated earlier as optimum effective dose for mammalian muscle cells mimicking in vivo conditions [10, 12, 21, 23]. We further titrated TNF‐α and IFN‐γ concentration to test a dose dependent effect. We reduced the original TNF‐α and IFN‐γ doses by half and one‐fourth of the original effective dose. With increasing dose of cytokines, we observed a trend i.e., in progressive loss of myotubes contraction accompanied by progressive reduction of MyHC‐IId expression (Figure S1A,B). The most prominent effect can be observed in previously establish effective dose of 10 ng/mL TNF‐α and 100 ng/mL IFNγ.

### 3.2 CIC Hampers Ca^2+^ Handling

**FIGURE 2:**
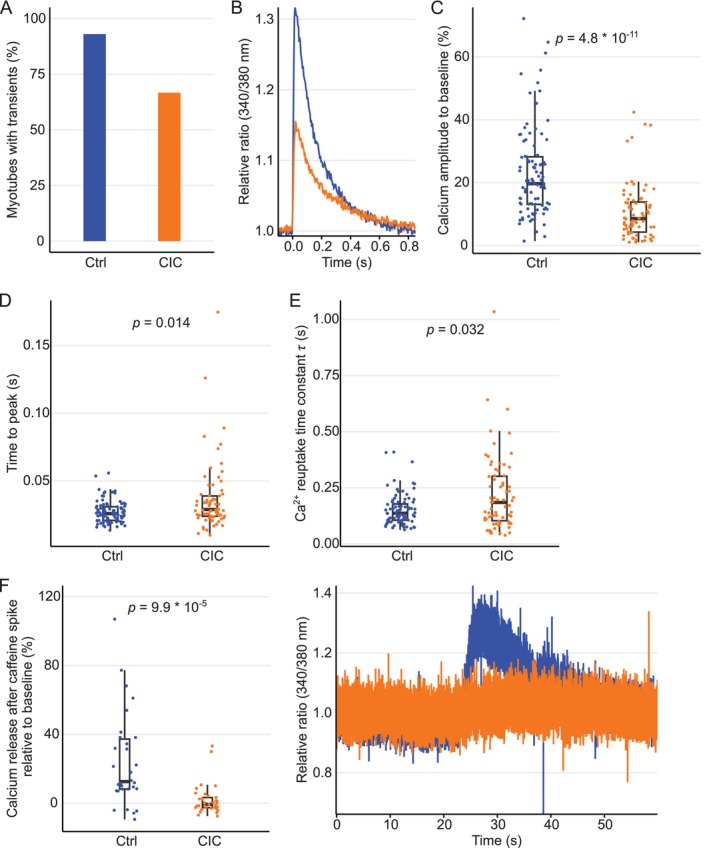
Cachexia impairs Ca^2+^ release and re‐uptake in skeletal myotubes. (A) Percentage of myotubes with an analysable transient. (B) Representative Ca^2+^ transients from individual Ctrl and CIC myotubes induced by electrical stimulation at 25 V, 1 Hz and 4 ms pulse duration. (C) Ca^2+^ amplitude (peak) compared to baseline in percentage, (D) time between electric stimulus until Ca^2+^ peak (maximal Ca^2+^ release) is reached in seconds, and (E) single exponential decay of Ca^2+^ relaxation time constant (τ) in seconds. (A)–(E) In total, we analysed 100 Ctrl and 108 CIC cells from four biological replicates. (F) Maximal released Ca^2+^ (amplitude) after addition of 10 μM caffeine to induce RyR gating (left panel). Data represent 30 Ctrl and 33 CIC myotubes from four biological replicates and a representative Ca^2+^ release from individual cells induced by caffeine (right panel). (C)–(F) Whisker plot shows median, first and third quartiles (hinges) and 1.5 interquartile ranges (whiskers), significance was tested using Mann–Whitney‐U‐test. Abbreviations: CIC: cancer secreted pro‐inflammatory cytokine‐induced cachexia, Ctrl: control.

The contractile property of muscle cells is a functional readout of acto‐myosin cross‐bridge cycling during ECC process. A proper Ca^2+^ release from and reuptake back to the SR is critical for muscle contraction and relaxation. Since CIC perturbed cell contraction, we asked if altered Ca^2+^ handling properties could be a cause for the impaired contraction of cachectic myotubes and probed the kinetics of intracellular Ca^2+^ transients in myotubes. Differentiated myotubes were loaded with the Ca^2+^ ion‐binding agent Fura‐2 AM, followed by electric stimulation. Corresponding to the stimulation frequency, the fluorescent signal traces showed a sharp rise in the fluorescent intensity above the baseline, followed by a gradual decrease in intensity. Only 67% of cachectic myotubes exhibited an analysable Ca^2+^ transient, in contrast to 93% control myotubes (Figure 2A). Examples of typical Ca^2+^ transients for control and cachectic myotubes are shown in Figure 2B. Comparison of cells that exhibited Ca^2+^ transients in both conditions showed more than two‐fold reduction in the signal amplitude for cachectic myotubes than the control cells (Figure 2B,C). Additionally, time to peak was increased for cachectic myotubes, suggesting that the Ca^2+^ was released significantly slower from 26 ± 0.5 ms in control to 29 ± 5.1 ms in CIC (Figure 2D). Reduction in cytosolic Ca^2+^ and re‐uptake of Ca^2+^ ions to the SR is necessary for muscle relaxation. This re‐entry of Ca^2+^ ions is governed by the sarcoendoplasmic Ca^2+^ ATPase pump SERCA1. The time constant of Ca^2+^ uptake (τ) in cachectic myotubes was significantly increased compared to control cells (Figure 2E), indicating a prolonged time of re‐uptake of Ca^2+^ (137 ± 13 ms in control to 185 ± 24 ms in CIC, *p* = 0.032). These data suggest that CIC affects both Ca^2+^ release into cytosol and re‐uptake processes of the SR.

The ryanodine receptors located in the SR membrane are responsible for Ca^2+^ release from the SR into the cytosol. Caffeine is an agonist of RyR1, promoting the opening of the channel and increasing intracellular Ca^2+^ levels [24]. We tested if the addition of caffeine can rescue the Ca^2+^ release defect in cachectic myotubes and improve the peak amplitude in Ca^2+^ transients. First, to confirm the state of cachexia in this experimental setup we quantified the percentage of myotubes exhibiting a Ca^2+^ transient prior to caffeine spike‐in. Approximately 90% of control and 58% CIC myotubes exhibited measurable Ca^2+^ transient (Figure S2A). Noteworthy, prior to caffeine treatment, the percentage of CIC cells with proper calcium transient in Figure S2A is similar to that of Figure 2A, further supporting our earlier observation presented in the Figure 2A. The addition of caffeine in our experimental setup led to a prominent spike and prolonged intracellular Ca^2+^ levels in control myotubes, as expected from the caffeine effect. Strikingly, no improved peak amplitude for Ca^2+^ release was observed for cachectic myotubes (Figure 2F and S2B), suggesting that CIC‐impaired RyR1 function could not be restored by an agonist.

In another experiment, to probe the possible Ca^2+^ leak in CIC myotubes, we measured the Fura‐2 loaded myotubes in the presence of 2 μM Tg without electrical pacing and in Ca^2+^ free extracellular media. In this set up, the majority of Ctrl (~74%) and CIC (~94.4%) myotubes (*N* = 5 biological replicates, *n* = 19 control myotubes and 18 CIC myotubes) did not show any spontaneous change in fluorescence signal that corresponds to cytosolic calcium, rather the signal remained unchanged throughout the recording period (Figure S2C–D). These experiments indicate the possibility of Ca^2+^ leak as a reason for the observed defective Ca^2+^ transients could be less likely in CIC myotubes under these conditions.

Together, these findings show that CIC affects Ca^2+^ handling properties on multiple levels in skeletal myotubes by affecting both Ca^2+^ release as well as re‐uptake.

### 3.3 Sarcomere Assembly is Perturbed in CIC

**FIGURE 3:**
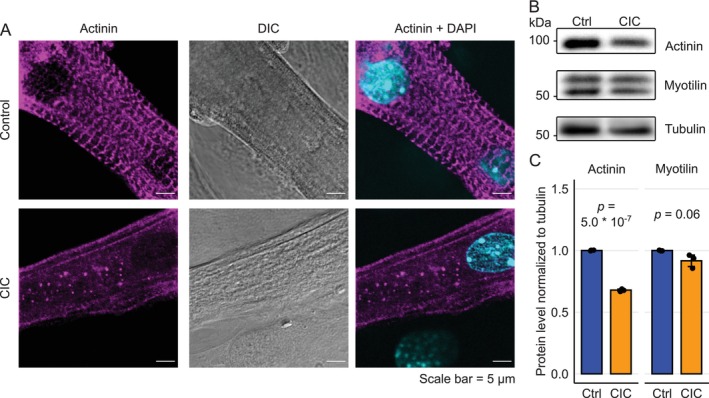
Sarcomeric disorganization in CIC. (A) Immunofluorescence staining against alpha‐actinin protein (marker for sarcomeric Z‐discs) in control and cachectic myotubes, scale bar is 5 μm. (B) Immunoblot of cell lysates stained against alpha‐actinin and myotilin, tubulin is shown as loading control. (C) Quantification of relative alpha‐actinin and myotilin protein expression from immunoblots normalized to tubulin. Data represents the mean ± SD from three biological replicates. Abbreviations: CIC: cancer secreted pro‐inflammatory cytokine‐induced cachexia, Ctrl: control, DAPI: 4′,6‐diamidino‐2‐phenylindol, DIC: differential interference contrast.

Despite impaired Ca^2+^ handling, 2/3rd of cachectic myotubes still exhibited a blunted Ca^2+^ transient (Figure 2A) and could induce some contraction. However, we did not identify any contracting myotubes in CIC (Figure 1C). These observations implied that in addition to Ca^2+^ homeostasis, other processes crucial for contraction might be targeted in CIC. Precisely organized sarcomere structure and proper function of sarcomeric components is key for myotube shortening and force generation. Therefore, we studied sarcomere organization in CIC. We stained paraformaldehyde‐fixed myotubes against the Z‐disc protein α‐actinin. In control myotubes, a well‐organized striated pattern, a hallmark of proper sarcomere assembly, was visible by laser scanning confocal microscopy. The characteristic sarcomeric striations were not observed in cachectic muscle cells (Figures 3A and S3). In CIC, α‐actinin showed statistically significant, but rather marginally reduced protein levels (reduced by ~32%), while myotilin, another Z‐disc protein, remained largely unchanged (Figure 3B,C). Altogether, CIC affected both SR‐related Ca^2+^ handling and sarcomere organization, culminating in loss of contraction.

### 3.4 CIC Alters Muscle Cell Proteostasis

**FIGURE 4:**
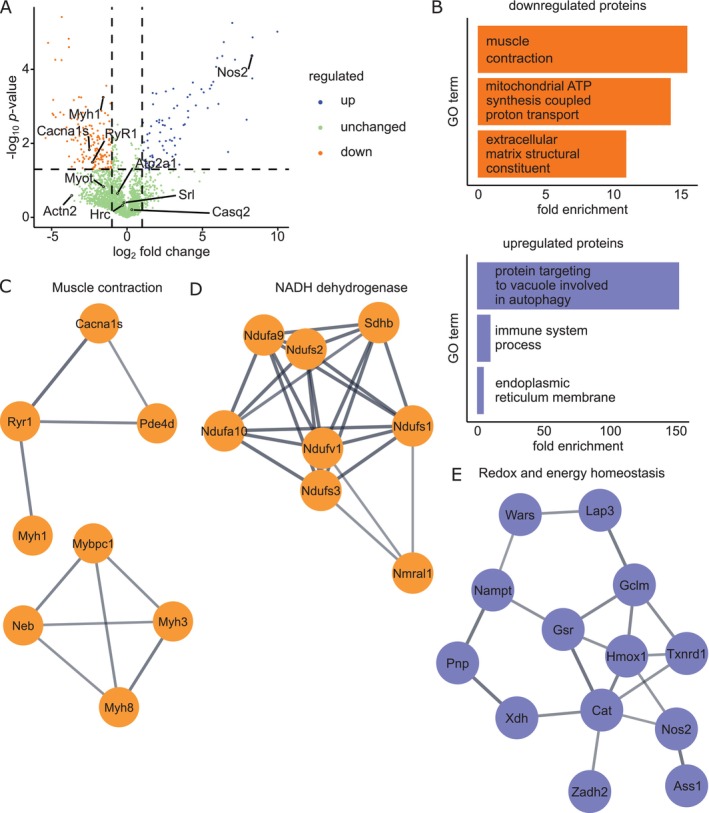
Quantitative mass spectrometric analysis of proteomic changes in cachectic myotubes. (A) Volcano plot showing log_2_ fold change of protein group (PG) expression levels and –log_10_ *p*‐value on x and y axis, respectively, PGs with a log_2_ fold change of 1.0 and a *p*‐value of 0.05 were considered significantly changed, statistical significance was tested using two‐way ANOVA, *N* = 3 biological replicates. (B) DAVID analysis showing selected enriched ontologies for down and up regulated PGs. (C)–(E) STRING analysis identifies clusters of PGs involved in (C) muscle contraction, (D) NADH dehydrogenase and (E) redox and energy homeostasis, which are altered in CIC.

To further understand detailed molecular mechanisms underlying CIC, we investigated differentially expressed proteins in cachectic vs. control cells using unbiased mass spectrometry‐based quantitative proteomic analysis. Control replicates showed a high correlation with each other, which was also true when comparing cachectic replicates with each other. However, the correlation between control and cachectic replicates was reduced (Figure S4A). Samples of the same condition clustered well together, and cachectic samples showed a strong distancing from control samples, as shown by principal component analysis (Figure S4B). These results indicated an overall reproducibility and high integrity outcome among independent experiments. Applying stringent filtering criteria resulted in 242 protein groups (PGs) with altered expression in CIC. Of these, 81 and 161 PGs were up‐ and down‐regulated in CIC, respectively (Figure 4A). Gene ontology (GO) enrichment analysis with DAVID [25] revealed that PGs with decreased expression were associated with terms for ‘muscle contraction’, ‘mitochondrial ATP synthesis coupled proton transport’, and ‘extracellular matrix structural constituent’. PGs with increased expression levels were enriched for ‘protein targeting to vacuole involved in autophagy’, ‘immune system process’, and ‘endoplasmic reticulum membrane’ (Figure 4B). By STRING (Search Tool for the Retrieval of Interacting Genes/Proteins, version 12.0) analysis, we identified two predominant clusters including proteins involved in contraction, i.e. Myh1/MyHC‐IId, RyR1, Cacna1s, and Mybpc1 (Figure 4C), for the down‐regulated PGs. Additionally, we identified another functionally interconnected cluster containing several subunits of the mitochondrial complex 1, the NADH dehydrogenase complex (Figure 4D). For up‐regulated PGs a functional cluster consisting of proteins involved in redox balance, like glutathione homeostasis, catalase, and nitric oxide synthase (Nos2) was identified (Figure 4E). Taken together, these findings suggest that CIC affects protein complexes involved in essential muscle functions, including muscle cell contraction and energy homeostasis.

### 3.5 CIC Alters Muscle Cell Transcriptome

**FIGURE 5:**
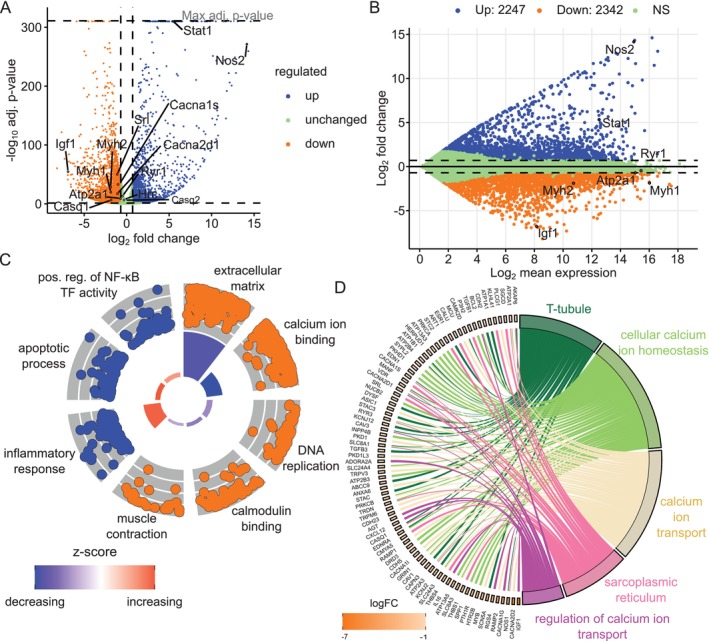
RNA‐sequencing identifies global changes in cachectic myotubes affecting Ca^2+^ handling, muscle contraction and inflammatory response. (A) Volcano plot showing log_2_ fold change of transcript expression level and –log_10_ *p* adj.‐value on x and y axis, respectively. Genes were considered as significantly changed with a log_2_ fold change of ≥0.7 and adj. *p*‐value of ≤0.05, from three independent experiments. B) MA plot shows the log_2_ fold change in relation to the log_2_ mean expression of each transcript, NS: not significant. (C) DAVID analysis reveals several enriched GO‐terms represented by a circle plot, where the dots in the outer circle represent relative expression change of a transcript. The inner circle with bars of different heights represent the –log_10_ *p* adj.‐value of the GO‐term and the colour indicates the z‐score of the term. Orange and blue colour in the outer circle represents decreased and increased transcripts, respectively. (D) Chord plot shows selected down regulated genes in CIC and their association with the indicated GO terms, the colour in the squares on the left half indicates the extent of log_2_ fold change in expression for each gene.

So far, it is unclear whether CIC has any general effect on the transcriptional output of muscle cells, which ultimately leads to an altered proteostasis. To test this, we performed RNA sequencing (RNA‐seq) experiments. Pearson correlation analysis revealed a high degree of inter‐sample correlation for transcript hits between biological replicates (Figure S5A). RNA‐seq identified 4589 de‐regulated genes in CIC. Of these, expression of 2247 genes was increased and 2342 genes were down‐regulated (Figure 5A). Noteworthy, myosin heavy chain (*Myh1* and *Myh2*) and *Atp2a1* (encoding SERCA1 protein) are among the significantly downregulated genes. Interestingly, *Ryr1* gene expression was not altered significantly, however two subunits of the DHPR, *Cacna1s* and *Cacna2d1*, were also down regulated at transcript level in CIC. Additionally, several Ca^2+^ storing proteins showed a reduced gene expression, i.e., sarcalumenin (*Srl*) and calsequestrin 1 (*Casq1*), but not the other paralog *Casq2* (Figure 5A). The de‐regulated genes, irrespective of expression level and their direction of change, are evenly distributed indicating a rather unbiased RNA‐seq analysis (Figure 5B). Furthermore, genes investigated in detail later in this study are located toward the higher end of expression, underlining their significance for muscle cell physiology. DAVID analysis revealed that down‐regulated genes primarily belong to processes related to ‘muscle contraction’ and ‘calcium ion binding’, among others (Figure 5C, Figure S5B). The chord plot analysis revealed that down‐regulated genes were assigned to the GO terms ‘T‐tubule’ and ‘SR’, with interconnected functions involved in biological processes to control ‘cellular calcium ion homeostasis’ and ‘calcium ion transport’ (Figure 5D). On the contrary, upregulated genes mainly belong to ‘inflammatory response’, ‘apoptotic process’, and ‘positive regulation of NF‐kB transcription factor activity’‐related processes (Figure 5C). Additionally, metascape analysis of our RNAseq data set indicated the NF‐kB pathway as among the most significantly activated regulatory pathways for up regulated genes in CIC (Figure S6A). Furthermore, we confirmed activation of NF‐κB by an increase in phosphorylated p65/RELA starting at 2 h of CIC induction (Figure S6B). Also, endoplasmic reticulum (ER) stress has been observed in conjunction with loss of RyR1 and as an underlying mechanism in several myopathies, such as CIC [26]. Therefore, we checked key marker genes involved in ER stress and found no significant up regulation of any ER stress markers (Figure S6C) from our RNAseq dataset. Furthermore, quantification of western blots from three different experiments showed that the ER stress marker GRP78 expression in CIC was at 65% of control (Ctrl) treatment levels (with a *p*‐value of 0.06). The other ER stress marker CHOP was expressed at 98% of Ctrl levels (with a *p*‐value of 0.72) (Figure S6D–E). These observations suggest that upregulation of ER stress pathway regulators, such as GRP78 and CHOP, is probably not among the major events in CIC in our experimental conditions. Collectively, whereas CIC reorients transcription of crucial genes such as *Myh1*/*2* and *Atp2a1* that are critically necessary for muscle contraction and calcium homeostasis, another key player, RyR1, was targeted by CIC at protein level.

### 3.6 Validation of Selected Targets Identified by MS and RNA‐seq at Transcript and Protein Levels

**FIGURE 6:**
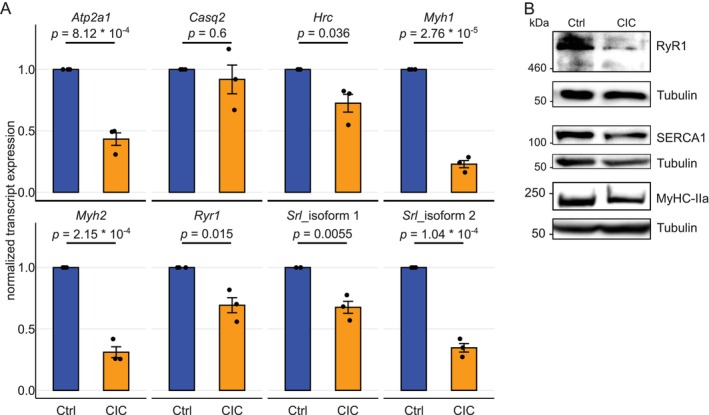
Confirmatory experiments show changes on transcript and protein level in CIC identified by MS and RNA‐seq. A) RT‐qPCR assays of indicated genes normalized to *Gapdh*, displayed as mean ± SEM from three biological experiments and nine technical replicates in total, significance was tested using two‐sided unpaired *t*‐test. (B) Immunoblot of cell lysates stained against RyR1, MyHC‐IIa, and SERCA1, Tubulin is shown as loading control, from *N* = 4 biological replicates. Abbreviations: CIC: cancer secreted pro‐inflammatory cytokine‐induced cachexia, Ctrl: control.

To further validate our mass spectrometry and RNA‐seq data we performed independent experiments and quantified changes on transcript and protein levels of selected candidates. Similar to our RNA‐seq results, in RT‐qPCR assays no changes in levels of *Casq2* and *Hrc* were observed. Consistent with our RNA‐seq, however, a strong down‐regulation of *Myh1* (~3‐fold), *Myh2* (~2.8‐fold), and *Atp2a1* (~2.3‐fold) transcripts was observed in CIC (Figure 6A). In line with this observation, we also detected lower levels of MyHC‐IId (gene *Myh1*) (Figure 1B), MyHC‐IIa (gene *Myh2*), and SERCA1 (gene *Atp2a1*) proteins in CIC condition (Figure 6B). For *Srl*, in line with our RNA‐seq analysis, we confirmed that the short isoform 2 was down regulated on transcript level (Figure 6A). Noteworthy, although changes of *Ryr1* transcript was rather marginal (i.e., 31%, or 1.2‐fold lowered in CIC), we detected a drastic (~68%) decline in RyR1 protein level (Figure 6B), suggesting that CIC likely targets RyR1 mainly at protein level.

Previously, several studies identified leaky RyR1 channels as an underlying cause of impaired Ca^2+^ release and thereby muscle weakness in myopathies [27, 28]. Under higher cellular redox state, post‐translational modification of RyR1 particularly by nitrosylation can disrupt interaction between RyR1 and the RyR1 stabilizing protein calstabin‐1, leading to leaky RyR1 channel. To investigate, whether unstable RyR1 could be contributing to the cachexia‐induced reduction in Ca^2+^ release (as was observed in Figure 2), we immunoprecipitated endogenous RyR1 and checked RyR1‐calstabin‐1 interaction as a read‐out of RyR1 modification in CIC. As RyR1 protein expression is reduced in cachectic myotubes, the intensity of immunoprecipitated RyR1 in cachectic eluates was decreased as well (Figure S7B). Then we probed immunoprecipitated fraction for calstabin‐1 and quantified the blots interaction between RyR1 and calstabin‐1. We did not identify a change in RyR1/calstabin‐1 interaction in CIC (Figure S7A–C). Furthermore, we found any change neither in whole cell nitrosylation, nor detected any RyR1 nitrosylation in cachectic conditions (Figure S7). These observations indicate that the reduced RyR1 protein expression is the main responsible factor for impaired Ca^2+^ release in CIC and that leaky channel due to nitrosylation is less likely to be a major contributing reason to this phenotype.

### 3.7 CIC Alters Active Epigenetic Marks and Chromatin Recruitment Of Transcriptionally Active RNA Polymerase II

Chromatin signalling precedes expression status of a given gene. Since CIC altered expression of distinct muscle‐specific genes, we tested if CIC plays any role in altering chromatin‐related transcriptional mechanisms. To investigate this, we established ChIP‐qPCR (chromatin immunoprecipitation‐qPCR) and designed promoter primer pairs to monitor chromatin‐related events of *Myh1* and *Atp2a1* genes. We focused on these genes as they were significantly downregulated and critical for muscle contraction and calcium transient processes.

**FIGURE 7:**
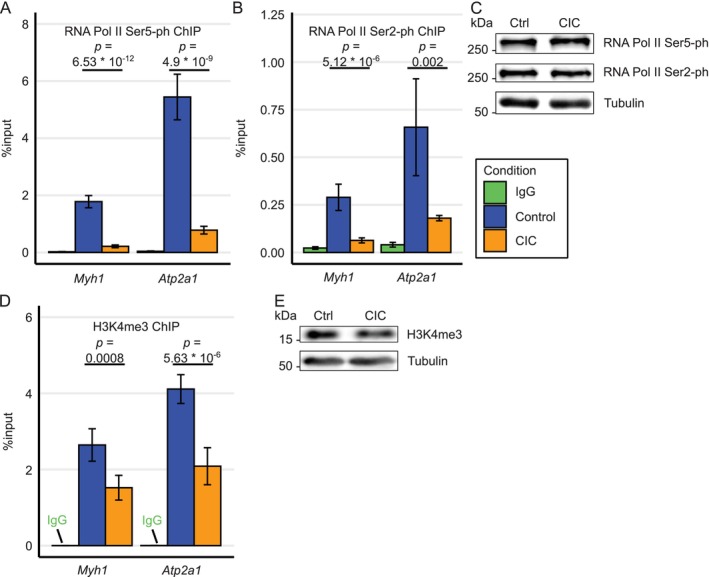
Transcriptionally active RNA polymerase II and H3K4me3 epigenetic marks are reduced on muscle‐specific genes in cachectic cells. ChIP assays were performed with indicated antibodies. (A) RNA Pol II Ser5‐ph (phosphorylated) and (B) RNA Pol II Ser2‐ph. (C) Immunoblots of total level of RNA Pol II Ser2‐ph and Ser5‐ph, tubulin is shown as a loading control. (D) Same as in (A) except, the ChIP assays were performed by using anti‐H3K4me3 antibody. (E) Immunoblot shows level of total H3K4me3 in Ctrl vs. CIC myotubes, tubulin is shown as a loading control. (A), (B) and (D), data represent the mean ± SEM from three biological replicates including technical triplicates in each experiment, significance was tested using two‐sided, unpaired *t*‐test. (C) and (E), representative blots from three biological replicates are shown. Abbreviations: CIC: cancer secreted pro‐inflammatory cytokine‐induced cachexia, Ctrl: control.

Association of transcriptionally active RNA polymerase II (Pol II) with chromatin domains denotes the active transcriptional state of a given gene. Phosphorylation of serine 5 and serine 2 (Ser5‐ph and Ser2‐ph) at the C‐terminal domain (CTD) of Pol II are known as transcriptionally active species of Pol II [29]. Since we observed decreases in muscle‐specific genes (including *Myh1* and *Atp2a1*), we sought to check association of Pol II on these genes. Our ChIP assays showed, compared to IgG ChIP control, a strong enrichment of both Ser2‐ph (12.5‐fold and 16.2‐fold on *Myh1* and *Atp2a1* promoters, respectively) and Ser5‐ph (76‐fold and 134‐fold on *Myh1* and *Atp2a1* promoters, respectively) in control myotubes (Figure 7A–B). In cachectic condition, chromatin recruitment of both Ser5‐ and Ser2‐phosphorylated species of Pol II were strongly reduced from the promoters of *Myh1* (3.7‐ and 4.4‐fold, respectively) and *Atp2a1* (6.9‐ and 8.5‐fold, respectively). Interestingly, the total protein levels of both phosphorylated forms of Pol II remain unaltered in CIC (Figure 7C).

Association of transcriptionally active Pol II to open chromatin domains could be because of increased active Histone 3 lysine 4 trimethylation (H3K4me3) mark on chromatin [30]. Thus, we argued if lower association of Pol II with *Myh1* and *Atp2a1* was actually caused by a change in H3K4me3 mark in CIC. To test this, we performed further ChIP assays to detect H3K4me3 marked nucleosomes. Compared to IgG control, qPCR experiments using ChIP‐enriched DNA templates from H3K4me3 ChIPs showed a strong enrichment (1700‐fold and 1100‐fold on *Myh1* and *Atp2a1*, respectively) of H3K4me3 marks. Interestingly, H3K4me3 mark was significantly reduced on both *Myh1* and *Atp2a1* promoters approx. 2‐fold in cachectic myotubes (Figure 7D). However, global H3K4me3 levels were unchanged in CIC (Figure 7E). Taken together, CIC decreased transcriptionally active marks and, consequently, chromatin recruitment of transcriptionally active Pol II was compromised on *Myh1* and *Atp2a1* genes. This serves as one of underlying causes as how CIC represses transcription of crucial muscle‐specific genes.

Previously, our lab identified the SUMO isopeptidases SENP3 and SENP7 as major regulators of *Myh1* expression by altering the chromatin landscape and these pathways were impaired in muscle atrophy [21, 31]. Therefore, we asked, whether these SENPs might have a regulatory role on *Atp2a1* expression as well. siRNA‐mediated knockdowns of SENP3 and SENP7 was confirmed by RT‐qPCR. The results show that SENP3 siRNA led to 4.4‐fold reduction in *SENP3* transcript and SENP7 siRNA led to 3.4‐fold decrease in *SENP7* transcript. Neither SENP3 nor SENP7 siRNA affected each other at transcript level, indicating specificity of these assays. Interestingly, in this set up, both SENP3 and SENP7 knockdown resulted in approximately two‐fold reduction of *Atp2a1* transcript levels (Figure S8A), indicating SENP3 and SENP7 are probably among the important upstream regulators in CIC. Noteworthy, SENP3 has been previously shown to regulate H3K4 trimethylation [32] and both SENP3 and SENP7 are required for proper RNA Pol II loading on its target genes including *Myh1* (*MyHC‐IId*) [21, 31]. Both SENP3 and SENP7 protein levels are reduced in CIC [21, 31]. We investigated if restoring SENP3 and SENP7 levels individually could ameliorate CIC condition. Interestingly, an improved expression of both SENP3 and SENP7 in CIC myotubes could partially‐ but significantly‐ rescue (~3‐fold and ~2.5‐fold, respectively) *Atp2a1* gene expression (Figure S8B). This observation underlines that SENP3 and SENP7‐governed molecular pathways have important role toward partial improvement of CIC conditions in our assay condition.

### 3.8 Cachexia Impedes Function of Primary Striated Muscle Cells

**FIGURE 8:**
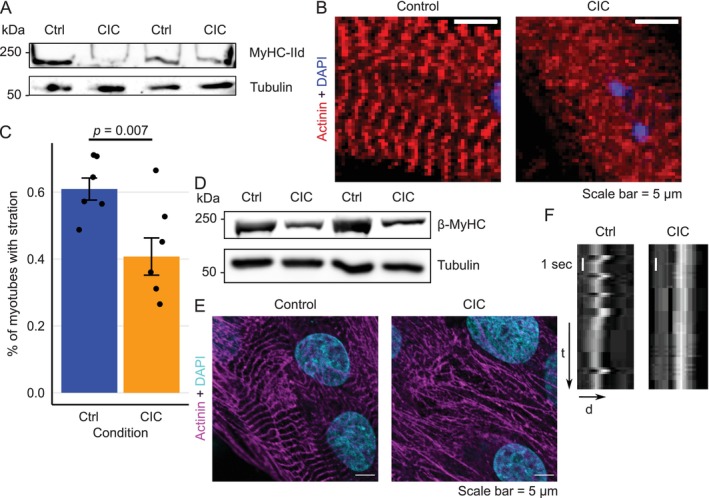
Cachexia severely disturbs contractility of primary myotubes. (A) Immunoblot of cell lysates from satellite stem cell (MuSC)‐derived primary muscle cells showing reduction of MyHC‐IId level in CIC, tubulin is shown as loading control. Representative blots from four individual mice per condition is shown. (B) Confocal images (immunofluorescence staining against alpha‐actinin) in control and cachectic primary muscle cells (treated with 10 ng/mL TNF‐α and 100 ng/mL INF‐γ for 48 h), scale bar is 5 μm. (C) Quantitative analysis of primary muscle cells with striation. Data was generated from six individual mice per condition; significance was tested using two‐sided *t*‐test. (D) Immunoblot of cell lysates from NRCMs showing reduced β‐MyHC in CIC, tubulin is shown as a loading control. Representative blot from three individual replicates is shown. (E) Same as in (B) except, NRCMs were used for these experiments. Representative images from three individual replicates is shown. (F) Kymograph comparing contracting NRCMs. Abbreviations: Ctrl: control, CIC: cancer secreted pro‐inflammatory cytokine‐induced cachexia.

To validate our findings, we performed few crucial experiments with mouse satellite cell derived primary muscle cells. In line with our observation in C2C12 progenitor cells‐derived myotubes, we also detected lowered MyHC‐IId protein level (Figure 8A) in primary muscle cells. Interestingly, we observed a significant decrease of cells in CIC (from 60% in Ctrl to 40% in CIC) with proper sarcomere organization (Figure 8B,C). Furthermore, neonatal rat cardiomyocytes (NRCMs) treated with TNF‐α and IFN‐γ again exhibited a strong reduction in β‐MyHC (the predominantly expressed myosin heavy chain isoform in cardiomyocytes) protein levels (Figure 8D) and disorganized sarcomeres in CIC (Figure 8E). In addition, the synchronous contraction of cardiomyocytes was disrupted in CIC (Figure 8F and Movie S2). These results indicate possible conserved regulatory mechanisms in both types of striated muscles that are deregulated in cachectic condition.

## 4 Discussion

In the current study, we demonstrate the multi‐pronged regulatory mechanisms underlying CIC. Through these mechanisms, primarily calcium transient pathway and sarcomere organization was found affected. One aspect of CIC was a more direct effect on protein levels of critical muscle‐specific regulators, including RyR1. We reason that a significant reduction in RyR1 protein levels in CIC, served as a major cause for a strongly blunted release of Ca^2+^ from the SR. Apart from Ca^2+^ release, Ca^2+^ reuptake was also found compromised as a result of reduced levels of Ca^2+^ pump SERCA. The transcriptomic analysis revealed that the effect on SERCA1 level is aggravated by the reduced gene expression. Besides, deregulated epigenetic and transcriptional mechanisms led to downregulation of genes, particularly *Myh1*, *Cacna1s*, and *Atp2a1* etc. in CIC. These multiple aspects of regulation ultimately culminated in impaired Ca^2+^ handling and loss of functional sarcomere structure leading to dysfunctional muscle cells that lacked contractile ability.

Cachexia is a whole‐body metabolic syndrome that remains poorly understood. CIC affects skeletal muscle in a plethora of ways, by deregulating energy homeostasis [33], catabolism/anabolism [34], and gene expression [35, 36] among others. A detailed mechanism by which muscle Ca^2+^ homeostasis is affected in CIC was unclear. Our proteomic data showed that RyR1 is targeted in CIC with an approximately 80% reduction at protein level. This, most likely, blocked Ca^2+^ release during ECC. Our Ca^2+^ transient experiment in presence of caffeine strongly supports this notion. Whereas caffeine, an agonist of RyR1 [24], led to a steep surge in Ca^2+^ release in control cells, the same resulted in no significant response in CIC. Furthermore, we provide evidence that neither depleted Ca^2+^ stores, nor leaky RyR1 channels contribute to impaired Ca^2+^ release in CIC (Figure S2C‐D and Figure S7). In addition to RyR1, DHPR expression was also down regulated in CIC (Figure 4C). The transverse tubules are highly organized sarcolemmal invaginations important for EC coupling. Here, four DHPR heterotetramers interact in a highly ordered manner with every other RyR1 homo‐tetramer located in the membrane of terminal cisternae [37]. Disturbing this stoichiometry might severely affect the transduction of action potentials toward the RyR1 and thereby Ca^2+^ release. Interestingly, in our study, the DHPR subunit α1s, which physically interacts with and activates RyR1 during EC coupling [1], was also reduced on protein level in CIC. Importantly, proper function of SERCA1 ensures rapid uptake of released Ca^2+^ into the SR during a single Ca^2+^ transient event. Thus, the key function of maintaining Ca^2+^ homeostasis by SERCA1 is crucial for muscle function and health. Our study revealed that Ca^2+^ reuptake to the SR is blunted in CIC, measured by longer single exponential decay (τ) of intracellular Ca^2+^ (Figure 2E). Taken together, our study showed that deregulation of multiple components of Ca^2+^ handling machinery is one of the key mechanisms prevalent in CIC. These components include DHPR, RyR1, as well as SERCA1 pump.

RyR1 depletion is a hallmark of several myopathies [26, 38, 39]. However, these reports did not investigate the role of RyR1 and SR Ca^2+^ handling in the context of cancer secreted cytokines (TNFα and IFNγ)‐induced cachexia. Here, we have demonstrated previously undetermined multipronged regulatory mechanisms underlying CIC. These mechanisms primarily target calcium transient pathway and sarcomere organization. In the context of RyR1, CIC predominantly altered RyR1 at protein level. Regarding MyHC‐IId and SERCA1, CIC altered chromatin signalling on the promoters of these genes (*Myh1, Atp2a1*) leading to reduced active transcription state and consequently lowered protein level of these regulators. The chromatin signalling includes changes in distinct epigenetic marks (H3K4 methylation) and impaired loading of transcriptionally active RNA Pol II on these genes (*Myh1, Atp2a1*) under CIC condition. Through these molecular mechanisms, CIC affects functionally related pathways in skeletal muscle cells including sarcomere organization and calcium transient process, ultimately impinged on muscle cell contraction. Our result further highlighted the complex nature of aetiology of cachexia.

The implications of higher intracellular Ca^2+^ are far‐reaching, as it is an important signalling molecule for many biological processes. During EC coupling, mitochondria take up Ca^2+^ ions that are released from the SR resulting in an increased ATP synthesis to fuel the energy demands of a contracting cell [40]. A disrupted Ca^2+^ cross‐talk between these organelles can have severe effects and contribute to the energetic imbalance commonly observed in CIC. Interestingly, we identified several upregulated proteins involved in redox homeostasis. On the other hand, several subunits of the mitochondrial respiratory chain complex I, NADH dehydrogenase, were specifically showing reduced protein levels, indicating a possible functional impediment of mitochondrial dynamics.

A prominent finding in this study is that, apart from direct effect on protein homeostasis, CIC suppresses the gene expression at the transcriptional level for distinct muscle‐specific genes, including *Myh1*, *Myh2*, *Atp2a1*, and *Cacna1s* etc. Mechanistically, CIC strongly precluded binding of transcriptionally active species of RNA Pol II on *Myh1* and *Atp2a1* promoters, indicating CIC can potentially affect active transcriptional processes. Further analysis revealed reduced levels of active epigenetic marker, i.e., histone modification at H3K4me3 on *Myh1* and *Atp2a1* promoters in CIC. Thus, it is conceivable that reduced level of H3K4me3 is the upstream event of impaired occupancy of active RNA Pol II. This notion is in line with previous findings that active RNA Pol II co‐occupies chromatin domain enriched in H3K4me3 marks [30]. Specifically, the recruitment of RNA Pol II and H3K4me3 appears to be deregulated in CIC for the muscle‐specific genes *Myh1* and *Atp2a1*, as Ser2‐ph and Ser5‐ph RNA Pol II protein level and global H3K4me3 signal remain largely unaltered (Figure 7C,E). At present, it is unclear if any further upstream event modulates H3K4me3 marks on muscle‐specific genes. One attractive hypothesis is functional alteration of histone methyltransferases, including SET1/MLL complex that catalyses H3K4me3 marks, in CIC. Additionally, we identified a putative role of SENP3 and SENP7 in *Atp2a1* regulation (Figure S8). Collectively, these data explained the mechanism that lowered transcriptional output of muscle‐specific genes in CIC, ultimately resulting in sarcomere disorganization and deregulated Ca^2+^ homeostasis.

In our current study, we used murine satellite cell derived primary muscle cells, primary neonatal rat cardiomyocytes and mouse C2C12 progenitor cell derived mature muscle cells (myotubes). Although these model systems served a key role in dissecting a detailed molecular mechanism underlying CIC, in vivo validation in cachectic patient biopsies or animal models needs to be addressed in futures studies. These combined strategies might help us to further comprehend the complexity of cachexia.

## 5 Conclusion

In conclusion, the results presented herein place the sarcomere contractile machinery and SR as important cellular compartments predominantly affected in cancer cytokine‐induced muscle wasting. The coordinated action of SR and sarcomere, primarily determines the muscle's force generating and load‐bearing function. A common denominator of this regulation is calcium signalling. Our study offered an understanding of intricate mechanisms by which SR‐related Ca^2+^ handling is affected in CIC. This knowledge provides a framework for future studies addressing questions to ameliorate CIC by particularly modulating calcium homeostasis mechanisms in striated muscle cells.

## Ethics Statement

All the experimental procedures were performed under the ethical approval of the Italian Ministry of Health and the Institutional Animal Care and Use Committee (authorization no. 83/2019‐PR and N. 127/2012‐A). The authors certify that they comply with the ethical guidelines for publishing in the *Journal of Cachexia, Sarcopenia and Muscle*: update 2019 [41].

## Conflicts of Interest

The authors declare no conflicts of interest.

## Supporting information


**Figure S1** Concentration‐dependent induction of CIC C2C12 myotubes were treated with the indicated concentrations of TNF‐α and IFN‐γ relative to the standard concentrations used. TNF‐α (10 ng/mL) and 100 ng/mL IFN‐γ was considered as 100% CIC dose. Half and one‐fourth of this dose corresponds to 50% CIC dose and 25% CIC dose, respectively. A) Percentage of contracting myotubes were placed under electrical stimulation at 40 V, 1 Hz and 4 ms pulse duration. Displayed is the mean ± SD from six individual coverslips (dots) from two biological replicates per condition. In total, 367 control, 298 25% CIC, and 313 50% CIC myotubes were analysed. For the comparison in the same graph, we used the data points for 100% CIC dose from Figure 1C. Statistical significance was tested using an unpaired two‐sided *t*‐test. B) Immunoblot of cell lysates showing reduction of MyHC‐IId in CIC, tubulin is shown as loading control.
**Figure S2. RyR1‐dependent Ca2+‐handling is impaired in cachectic myotubes.** A) The same as in Figure 2A), but for cells analysed in Figure 2F) prior to caffeine‐induced Ca2+ release. Thirty Ctrl and 33 CIC myotubes were analysed from four biological replicates. B) Representative Ca2+ signals from individual Ctrl and CIC myotubes induced by 10 μM caffeine. C–D) Fura2‐loaded cells were placed in a custom‐made chamber with Ca2+‐free extracellular perfusion solution at 37°C. Ca2+ transient measurements were started immediately after keeping the cells in Ca2 + −free extracellular solution without pacing. After 60 s of recording, 2 μM Thapsigargin (Tg) was added to the Ca2+‐free extracellular solution and recorded (without pacing) for a further 120 s. Red arrow indicates time when Tg was added. Red line indicates Ca2+ measurement period in presence of Tg. Representative Ca2+‐traces of myotubes are shown. *N* = 5 biological replicates. Majority of both Ctrl (C) and CIC (D) myotubes did not show any spontaneous change in fluorescence signal that corresponds to cytosolic Ca2+.
**Figure S3. Confocal images show disturbed sarcomeric organization in CIC.** The same as in Figure 3A, except, lower magnification images show disorganized sarcomere structures in CIC. Scale bar is 20 μm, DAPI: 4′,6‐diamidino‐2‐phenylindol, DIC: differential interference contrast. Images are representative of at least three independent experiments.
**Figure S4. Quality control of mass spectrometric analysis.** A) Multi‐scatter plot compares protein group expression profiles between all samples analysed by mass spectrometric quantitative proteomics approach. B) Principal component analysis shows distinct clustering for samples of the same condition. Data is generated from three independent experiments.
**Figure S5. Gene expression analysis of specific gene ontology terms.** A) Pearson correlation plot shows sample similarity based on log2‐normalized CPM (counts per million mapped reads) values. B) Gene expression changes (Figure 5) of genes assigned to the GO terms ‘muscle contraction’ and ‘regulation of muscle cell differentiation’, only genes with significantly changed expression levels in CIC are displayed. Data is generated from three independent experiments.
**Figure S6. NF‐κB and endoplasmic reticulum stress related gene expression in CIC.** A) TRRUST (transcriptional regulatory relationships unravelled by sentence‐based text‐mining) analysis generated using metascape platform of up regulated genes in CIC identifies several up regulated transcriptional pathways (y‐axis). Statistical significance of the regulated pathway is displayed on the x‐axis as −log10 *p*‐value. B) Immunoblot of p65 and p65 phosphorylated at Ser536 after 1, 2, and 4 h of induction of CIC. Tubulin is shown as a loading control (*N* = 3 biological replicates). C) Gene expression changes of key genes related to endoplasmic reticulum stress. RNA‐seq data presented in Figure 5 was analysed (*N* = 3 biological replicates). D) Immunoblot of GRP78 and CHOP in control and CIC myotubes. Tubulin is shown as a loading control (*N* = 3 biological replicates). E) Quantification of relative GRP78 and CHOP protein expression from immunoblots normalized to tubulin. Data represents mean ± SD from three biological replicates, including the blot presented in Figure S6D. Statistical significance was tested using two‐sided unpaired *t*‐test.
**Figure S7. RyR1 interaction with Calstabin‐1 is unchanged in CIC.** A) Cell lysates (input control) are probed with the indicated antibodies. B) RyR1 was immunoprecipitated from Ctrl and CIC myotubes followed by immunoblot analysis for calstabin‐1 and nitrosylation. All panels were originated from the same elute fraction. IgG antibody was used as a negative control for immunoprecipitation. C) Quantification of Calstabin‐1 to RyR1 ratio in the eluates displayed as mean ± SD from three biological replicates, significance was tested using two‐sided unpaired t‐test.
**Figure S8. SENP3 and SENP7 regulate *Atp2a1* expression in skeletal myotubes** A) RT‐qPCR assay of indicated genes normalized to *Gapdh* from myotubes transfected with indicated siRNAs, displayed as mean ± SEM from three biological experiments and nine technical replicates in total, significance was tested using two‐sided unpaired t‐test. B) RT‐qPCR assay of indicated genes normalized to *Gapdh* from myotubes transfected with 1 μg of either vector control or mouse SENP3 or SENP7 plasmids prior to induction of CIC. Data displayed as mean ± SEM from two biological experiments with technical quadruplicates in each experiment. Statistical significance was tested using two‐sided unpaired t‐test. CIC was same as in Figure 1A.

**Movie S1** **Contractile ability of control and cachectic myotubes.** The reaction of myotubes on coverslips immersed in differentiation medium and electrically paced at 40 V, 1 Hz, and 4 ms pulse duration was recorded at 15 frames/s. Movie is played at 20 frames/s. Scale bar corresponds to distance of 50 μm. The myotubes are pseudocoloured. Control (left) and CIC (right) cells are shown.

**Movie S2** **Contractile ability of control and cachectic cardiomyocytes.** The spontaneous contraction of cardiomyotubes on coverslips immersed in differentiation medium was recorded at 15 frames/s. Movie is played at 20 frames/s. Scale bar corresponds to distance of 50 μm. The myotubes are pseudocoloured. Control (left) and CIC (right) cells are shown.

**Table S1** **Processed data from mass spectrometric proteomics analysis.** Data after analysis with MaxQuant and Persues is shown. Significantly up‐ and downregulated protein groups are highlighted in blue orange, respectively.

**Table S2** **Processed data from RNA‐sequencing analysis.** Data after analysis with Galaxy is shown. Significantly up‐ and downregulated protein groups are highlighted in blue and orange, respectively.
