# Supplementary material for: Calcium Handling Machinery and Sarcomere Assembly are Impaired Through Multipronged Mechanisms in Cancer Cytokine‐Induced Cachexia
Source: J Cachexia Sarcopenia Muscle. 2025 Apr 4;16(2):e13776. doi: 10.1002/jcsm.13776 (PMC11969248; doi:10.1002/jcsm.13776)
Supplement: Supplementary file 1 — Figure S1 Concentration‐dependent induction of CIC C2C12 myotubes were treated with the indicated concentrations of TNF‐α and IFN‐γ relative to the standard concentrations used. TNF‐α (10 ng/mL) and 100 ng/mL IFN‐γ was considered as 100% CIC dose. Half and one‐fourth of this dose corresponds to 50% CIC dose and 25% CIC dose, respectively. A) Percentage of contracting myotubes were placed under electrical stimulation at 40 V, 1 Hz and 4 ms pulse duration. Displayed is the mean ± SD from six individual coverslips (dots) from two biological replicates per condition. In total, 367 control, 298 25% CIC, and 313 50% CIC myotubes were analysed. For the comparison in the same graph, we used the data points for 100% CIC dose from Figure 1C. Statistical significance was tested using an unpaired two‐sided t‐test. B) Immunoblot of cell lysates showing reduction of MyHC‐IId in CIC, tubulin is shown as loading control. Figure S2. RyR1‐dependent Ca2+‐handling is impaired in cachectic myotubes. A) The same as in Figure 2A), but for cells analysed in Figure 2F) prior to caffeine‐induced Ca2+ release. Thirty Ctrl and 33 CIC myotubes were analysed from four biological replicates. B) Representative Ca2+ signals from individual Ctrl and CIC myotubes induced by 10 μM caffeine. C–D) Fura2‐loaded cells were placed in a custom‐made chamber with Ca2+‐free extracellular perfusion solution at 37°C. Ca2+ transient measurements were started immediately after keeping the cells in Ca2 + −free extracellular solution without pacing. After 60 s of recording, 2 μM Thapsigargin (Tg) was added to the Ca2+‐free extracellular solution and recorded (without pacing) for a further 120 s. Red arrow indicates time when Tg was added. Red line indicates Ca2+ measurement period in presence of Tg. Representative Ca2+‐traces of myotubes are shown. N = 5 biological replicates. Majority of both Ctrl (C) and CIC (D) myotubes did not show any spontaneous change in fluorescence signal that corresponds to cytosolic Ca2+. [file JCSM-16-e13776-s003.pdf]

Supplemental information

Calcium handling machinery and sarcomere assembly are impaired through multipronged mechanisms in cancer cytokine-induced cachexia

Luis Vincens Gand[1], Chiara Lanzuolo[2,3], Mugeng Li[1], Valentina Rosti[2,3], Natalie Weber[4], Dongchao Lu[4], Christian Bär[4], Thomas Thum[4], Andreas Pich[5], Theresia Kraft[1], Mamta Amrute-Nayak[1]* and Arnab Nayak[1]*

This supplemental information contains (1) supplementary figures with legends, (2) supplementary movie and table legends, (3) supplementary materials and methods, and (4) supplementary references.

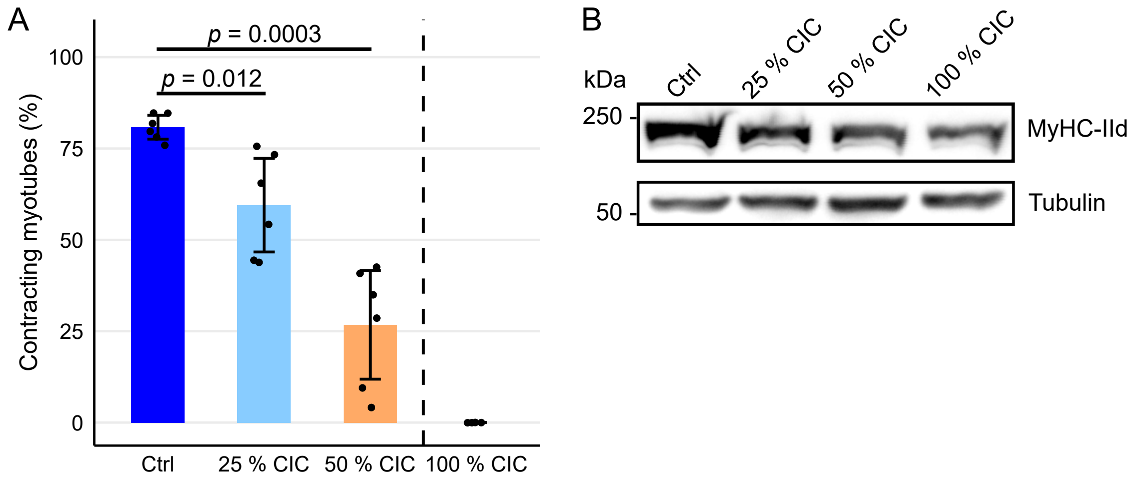

Figure S1 Concentration-dependent induction of CIC

C2C12 myotubes were treated with the indicated concentrations of TNF-α and IFN-γ relative to the standard concentrations used. 10 ng/ml TNF-α and 100 ng/ml IFN-γ was considered as 100 % CIC dose. Half and one fourth of this dose corresponds to 50 % CIC dose and 25 % CIC dose, respectively. A) Percentage of contracting myotubes were placed under electrical stimulation at 40 V, 1 Hz and 4 ms pulse duration. Displayed is the mean ± SD from 6 individual coverslips (dots) from 2 biological replicates per condition. In total, 367 control, 298 25 % CIC, and 313 50 % CIC myotubes were analyzed. For the comparison in the same graph, we used the data points for 100 % CIC dose from figure 1C. Statistical significance was tested using an unpaired two-sided t-test. B) Immunoblot of cell lysates showing reduction of MyHC-IId in CIC, tubulin is shown as loading control.

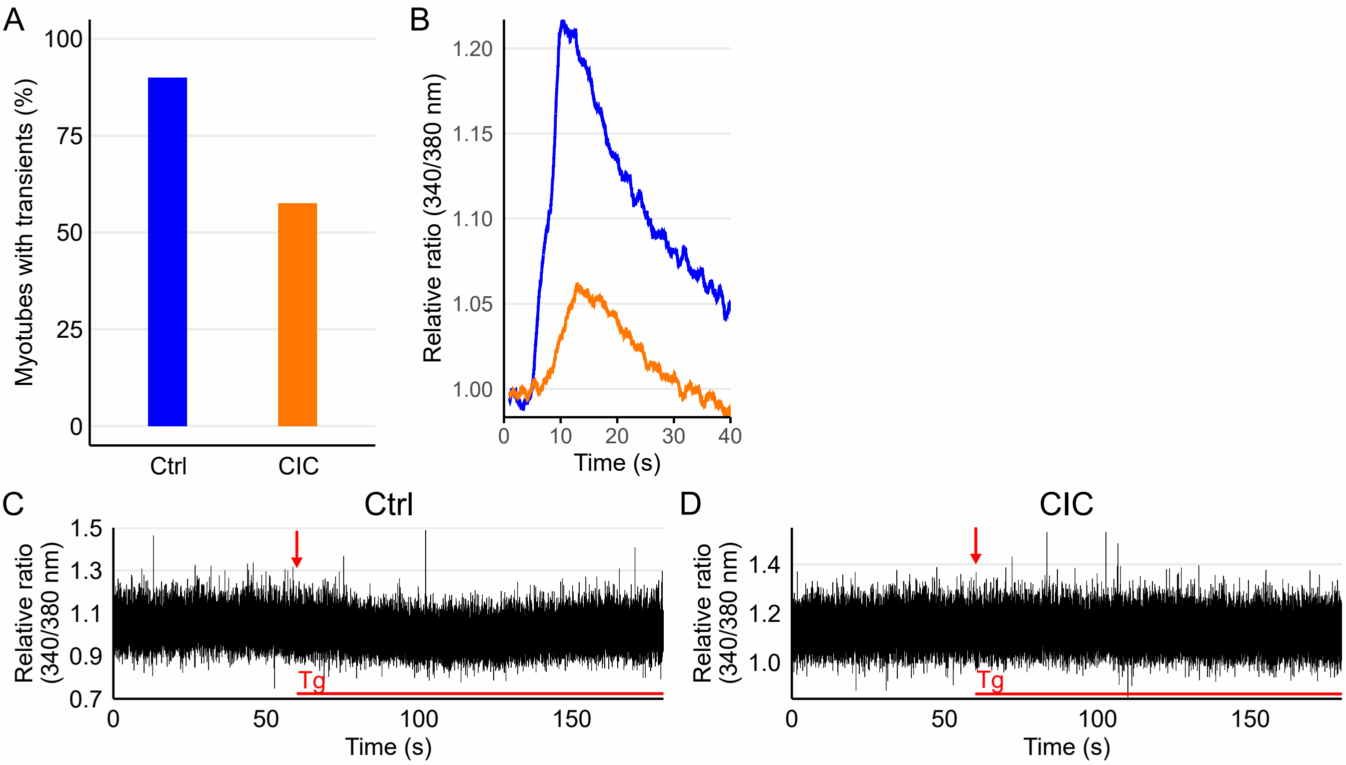

Figure S2 RyR1-dependent Ca^{2+}-handling is impaired in cachectic myotubes

A) Same as in Fig 2A), but for cells analyzed in Fig 2F) prior to caffeine-induced Ca^{2+} release. 30 Ctrl and 33 CIC myotubes were analyzed from 4 biological replicates. B) Representative Ca^{2+} signals from indivicual Ctrl and CIC myotubes induced by 10 μM caffeine. C-D) Fura2-loaded cells were placed in a custom-made chamber with Ca^{2+}-free extracellular perfusion solution at 37 °C. Ca^{2+} transient measurements were started immediately after keeping the cells in Ca^{2+}-free extracellular solution without pacing. After 60 sec of recording, 2 μM Thapsigargin (Tg) was added to the Ca^{2+}-free extracellular solution and recorded (without pacing) for a further 120 sec. Red arrow indicates time when Tg was added. Red line indicates Ca^{2+} measurement period in presence of Tg. Representative Ca^{2+}-traces of myotubes are shown. N = 5 biological replicates. Majority of both Ctrl (C) and CIC (D) myotubes did not show any spontaneous change in fluorescence signal that corresponds to cytosolic Ca^{2+}.

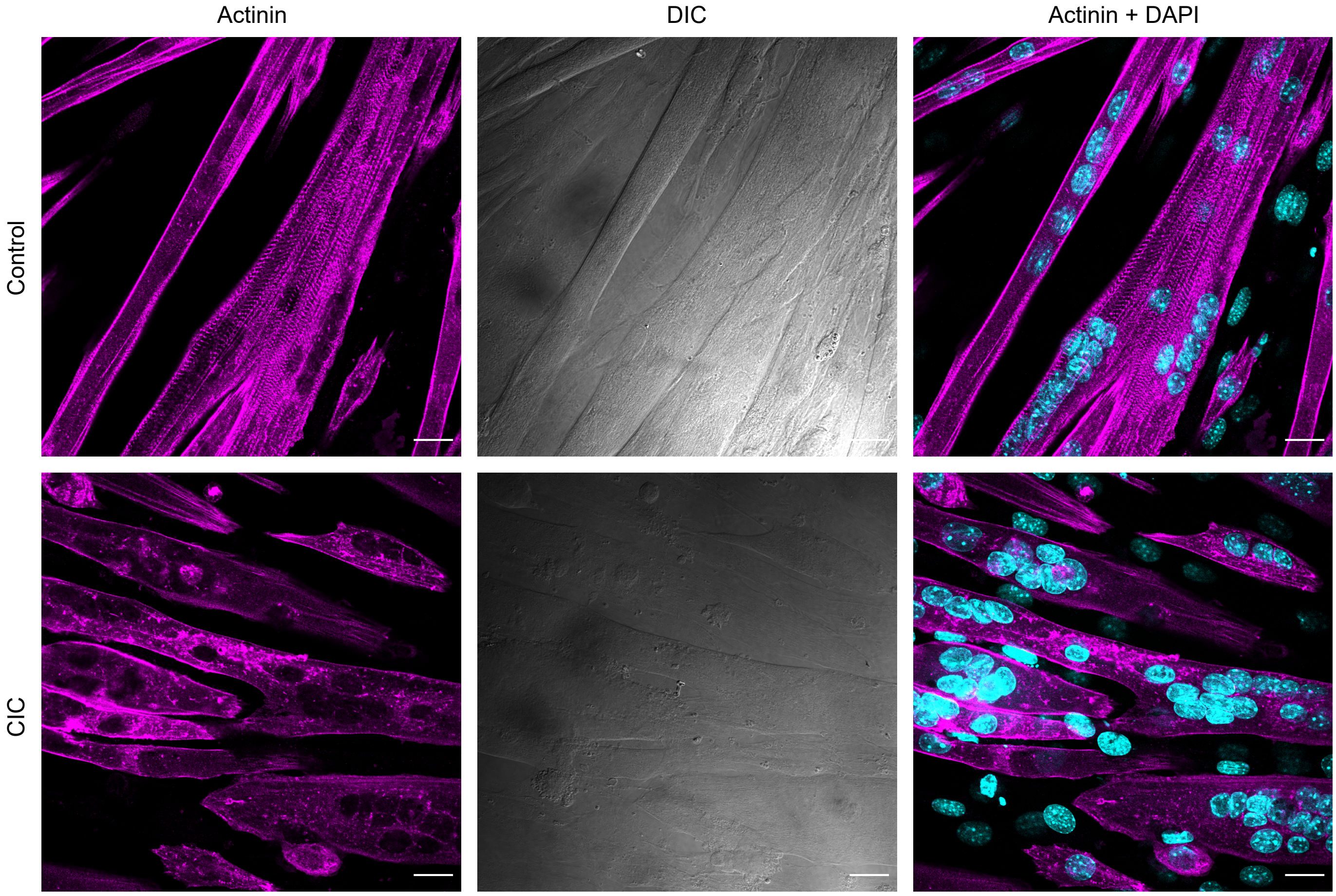

Scale bar = 20 μm

Figure S3 Confocal images show disturbed sarcomeric organization in CIC
Same as in Figure 3A, except, lower magnification images show disorganized sarcomere structures in CIC. Scale bar is 20 μm, DAPI: 4',6-diamidino-2-phenylindol, DIC: differential interference contrast. Images are representative of at least three independent experiments.

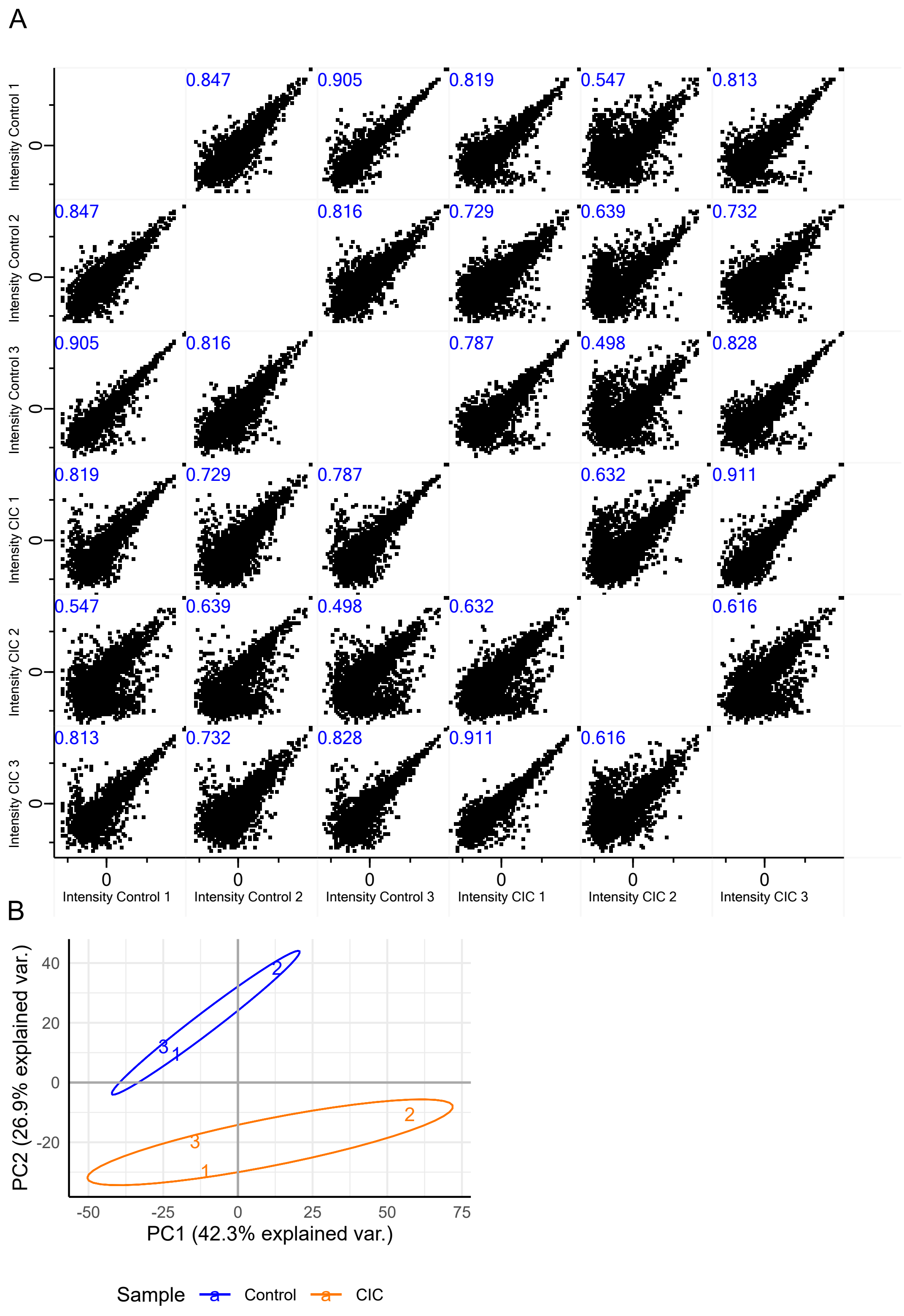

Figure S4 Quality control of mass spectrometric analysis

A) Multi-scatter plot compares protein group expression profiles between all samples analyzed by mass spectrometric quantitative proteomics approach. B) Principal component analysis shows distinct clustering for samples of the same condition. Data is generated from three independent experiments.

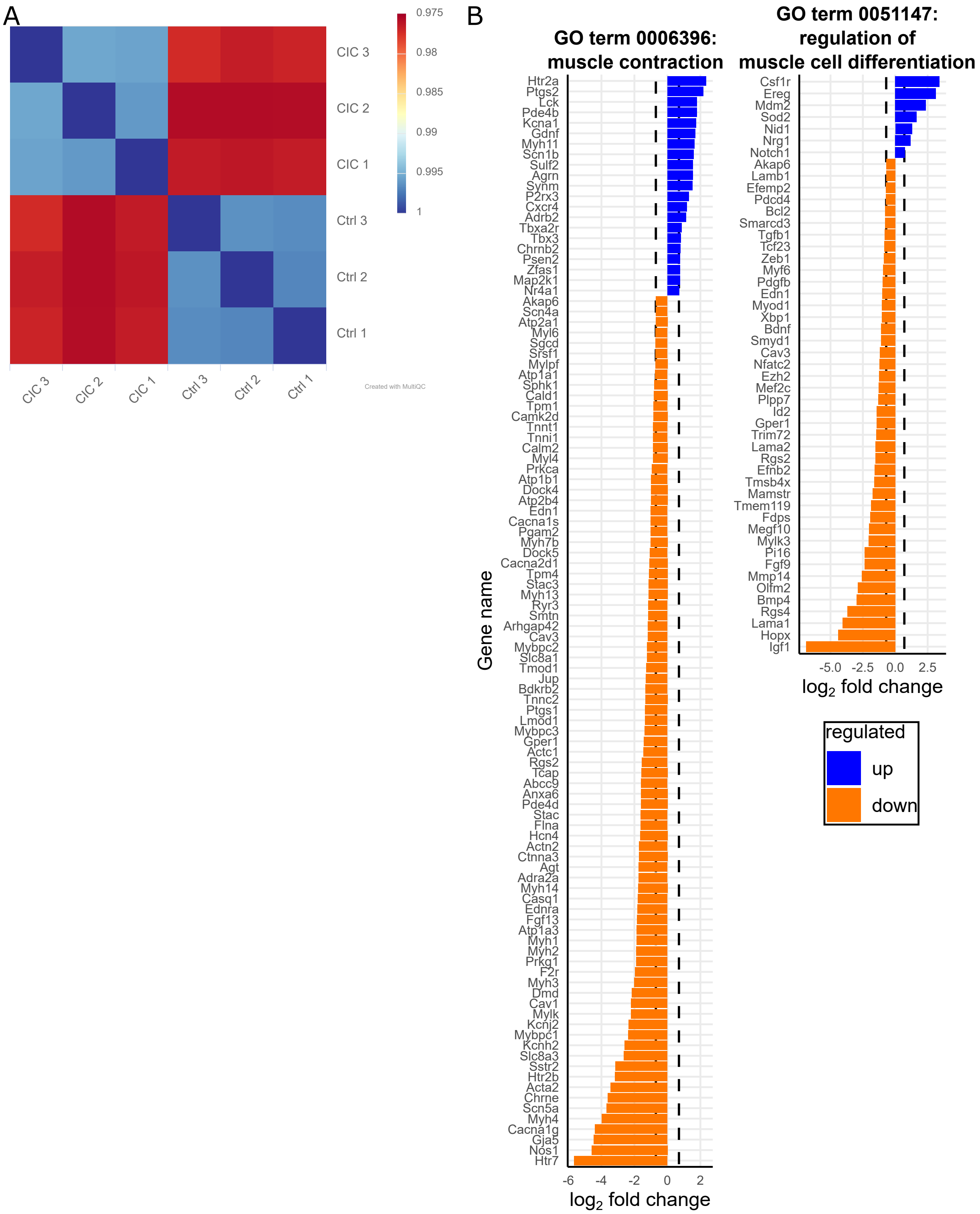

Figure S5 Gene expression analysis of specific gene ontology terms

A) Pearson correlation plot shows sample similarity based on log_2-normalized CPM (counts per million mapped reads) values. B) Gene expression changes (Figure 5) of genes assigned to the GO terms ‘muscle contraction’ and ‘regulation of muscle cell differentiation’, only genes with significantly changed expression levels in CIC are displayed. Data is generated from three independent experiments.

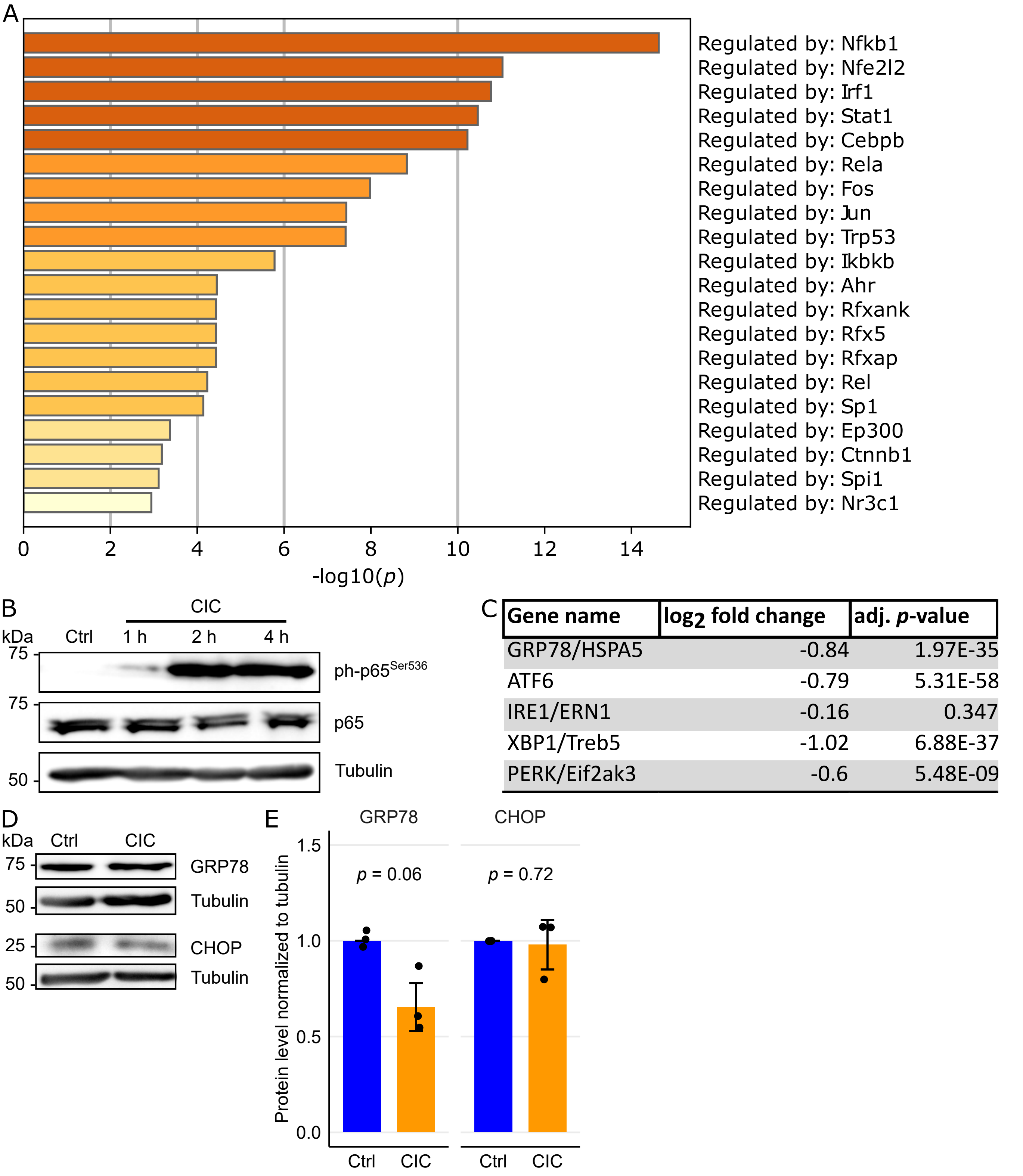

Gene name	log_2 fold change	adj. *p*-value
GRP78/HSPA5	-0.84	1.97E-35
ATF6	-0.79	5.31E-58
IRE1/ERN1	-0.16	0.347
XBP1/Treb5	-1.02	6.88E-37
PERK/Eif2ak3	-0.6	5.48E-09

Figure S6 NF-κB and endoplasmic reticulum stress related gene expression in CIC
A) TRRUST (transcriptional regulatory relationships unraveled by sentence-based text-mining) analysis generated using metascape platform of up regulated genes in CIC identifies several up regulated transcriptional pathways (y-axis). Statistical significance of the regulated pathway is displayed on the x-axis as $-\log_{10}$ *p*-value. B) Immunoblot of p65 and p65 phosphorylated at Ser536 after 1, 2, and 4 h of induction of CIC. Tubulin is shown as a loading control (N = 3 biological replicates). C) Gene expression changes of key genes related to endoplasmic reticulum stress. RNA-seq data presented in Fig. 5 was analyzed (N = 3 biological replicates). D) Immunoblot of GRP78 and CHOP in control and CIC myotubes. Tubulin is shown as a loading control (N = 3 biological replicates). E) Quantification of relative GRP78 and CHOP protein expression from immunoblots normalized to tubulin. Data represents mean ± SD from three biological replicates, including the blot presented in Figure S6D. Statistical significance was tested using two-sided unpaired t-test.

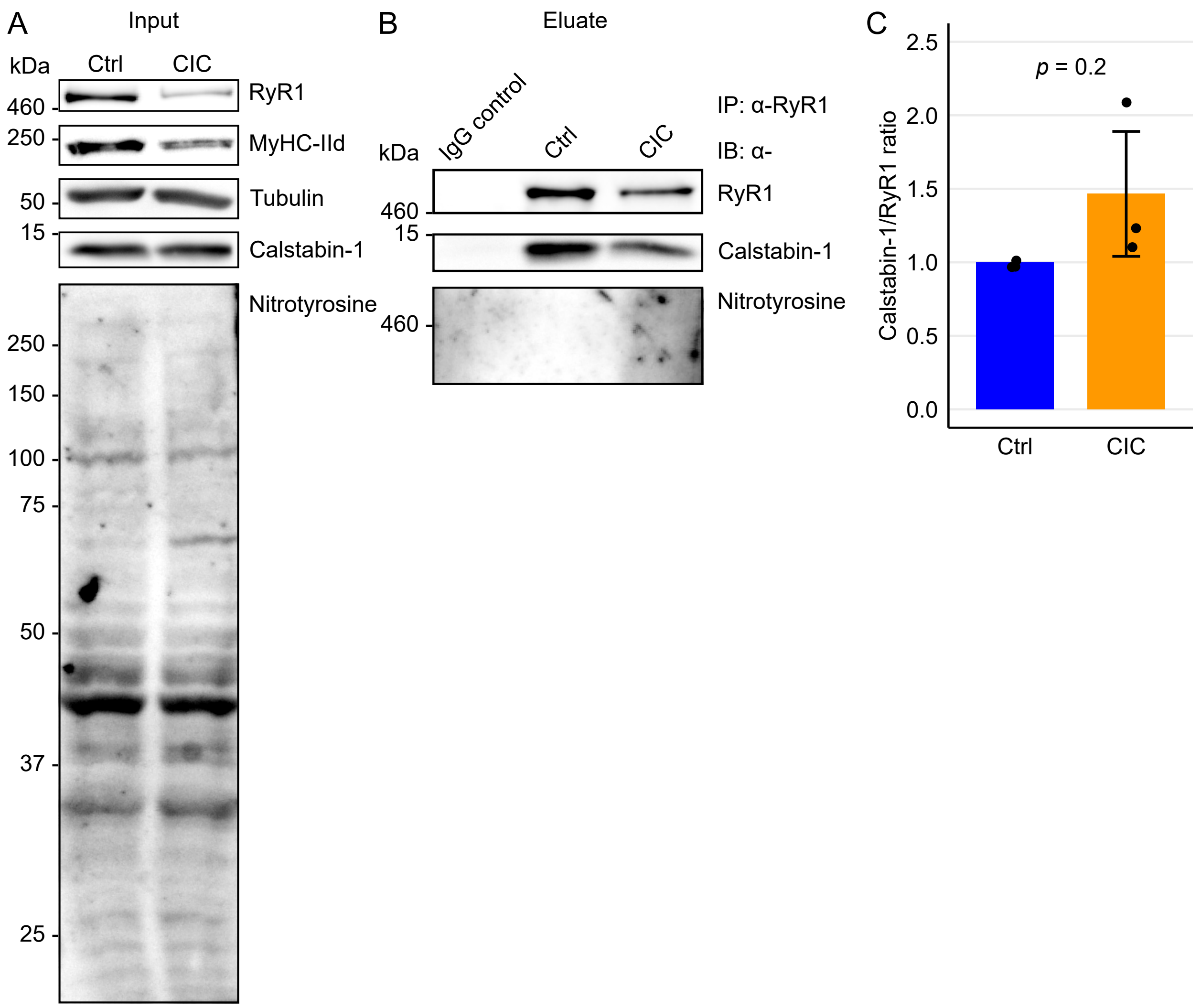

Figure S7 RyR1 interaction with Calstabin-1 is unchanged in CIC

A) Cell lysates (input control) are probed with the indicated antibodies. B) RyR1 was immunoprecipitated from Ctrl and CIC myotubes followed by immunoblot analysis for calstabin-1 and nitrosylation. All panels were originated from the same elute fraction. IgG antibody was used as a negative control for immunoprecipitation. C) Quantification of Calstabin-1 to RyR1 ratio in the eluates displayed as mean ± SD from 3 biological replicates, significance was tested using two-sided unpaired t-test.

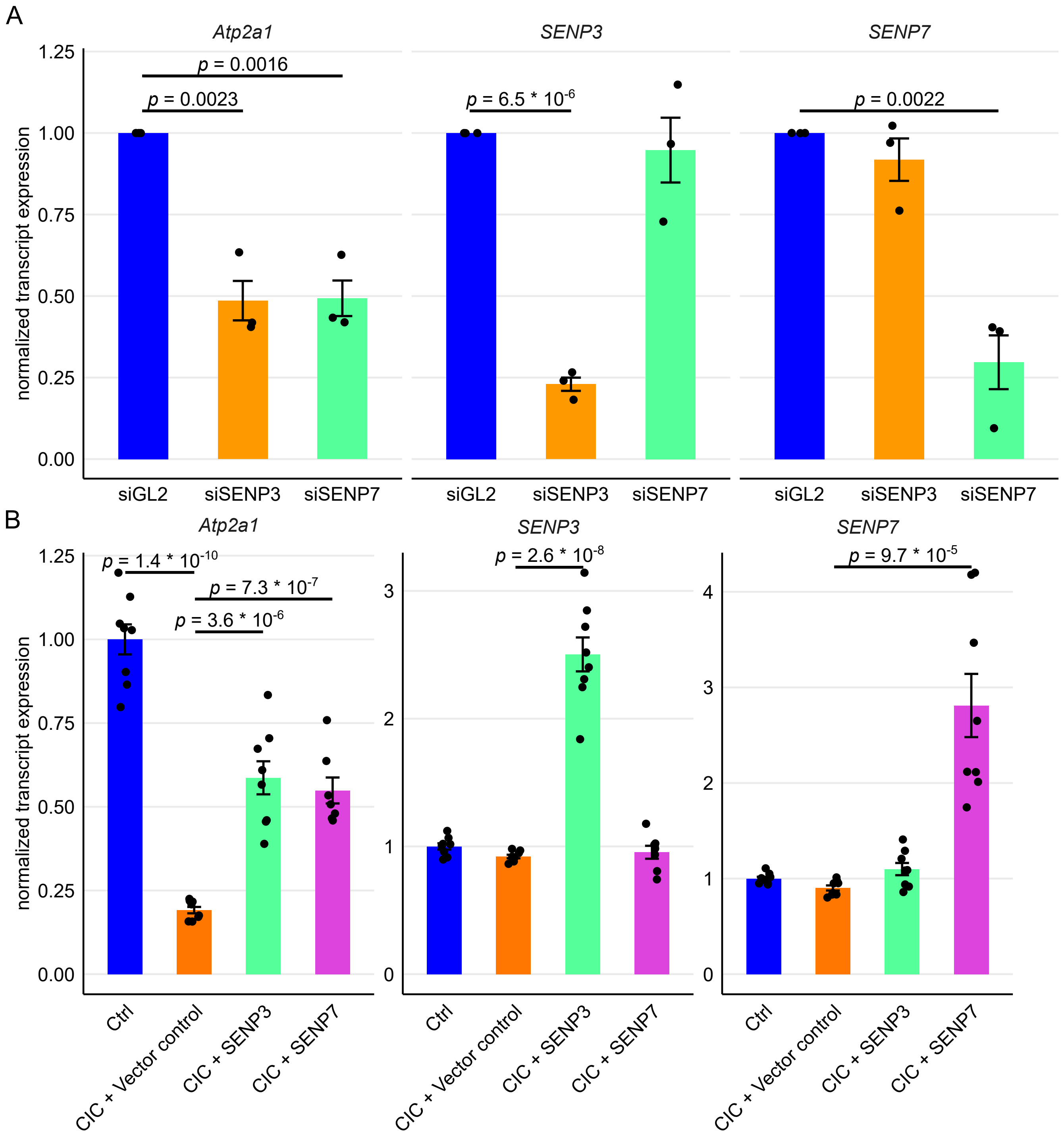

Figure S8 SENP3 and SENP7 regulate *Atp2a1* expression in skeletal myotubes
A) RT-qPCR assay of indicated genes normalized to *Gapdh* from myotubes transfected with indicated siRNAs, displayed as mean ± SEM from 3 biological experiments and 9 technical replicates in total, significance was tested using two-sided unpaired t-test. B) RT-qPCR assay of indicated genes normalized to *Gapdh* from myotubes transfected with 1 µg of either vector control or mouse SENP3 or SENP7 plasmids prior to induction of CIC. Data displayed as mean ± SEM from 2 biological experiments with technical quadruplicates in each experiment. Statistical significance was tested using two-sided unpaired t-test. CIC was same as in Figure 1A.

Supplementary movie and table legends

Supplementary movie S1 Contractile ability of control and cachectic myotubes

The reaction of myotubes on coverslips immersed in differentiation medium and electrically paced at 40 V, 1 Hz, and 4 ms pulse duration was recorded at 15 frames/sec. Movie is played at 20 frames/sec. Scale bar corresponds to distance of 50 µm. The myotubes are pseudocoloured. Control (left) and CIC (right) cells are shown.

Supplementary movie S2 Contractile ability of control and cachectic cardiomyocytes

The spontaneous contraction of cardiomyotubes on coverslips immersed in differentiation medium was recorded at 15 frames/sec. Movie is played at 20 frames/sec. Scale bar corresponds to distance of 50 µm. The myotubes are pseudocoloured. Control (left) and CIC (right) cells are shown.

Supplementary table S1 Processed data from mass spectrometric proteomics analysis

Data after analysis with MaxQuant and Persues is shown. Significantly up- and downregulated protein groups are highlighted in blue orange, respectively.

Supplementary table S2 Processed data from RNA-sequencing analysis

Data after analysis with Galaxy is shown. Significantly up- and downregulated protein groups are highlighted in blue and orange, respectively.

Supplementary material and methods

Antibodies

For western blotting the following antibodies were used: α-MyHC-IId (1:2,000; Abcam, UK, cat. no. ab91506), α-MyHC-IIa (1:1,000, Santa Cruz Biotechnology, USA, cat. no. sc-53095), α-SERCA1 (1:1,000; Invitrogen, USA, cat. no. MA3-912), α-RyR1 (1:1,000; Cell signaling, USA, cat. no. 8153S), α-ACTN2 (1:1,000, LSBio, USA, cat. no. LS-C351789), α-myotilin (1:700, Santa Cruz Biotechnology, USA, cat. no. sc-393958), α-MyHC-β (1:1,000, Sigma, USA, cat. no. M8421), α-RNA polymerase II serine 5 phosphorylation (1:1,000, Abcam, UK, cat. no. ab5131), RNA polymerase II serine 2 phosphorylation (1:1,000, Abcam, UK, cat. no. ab5095), α-H3K4me3 (1:1,000, Cell signaling, USA, cat. no. 9751), α-Calstabin-1 (1:500, Santa Cruz Biotechnology, cat. no. sc-133067), α-nitrosylation (1:500, Sigma-Aldrich, cat. no. N0409), α-RELA/P65 (Santa Cruz Biotechnology, cat. no. sc-8008), α-phospho-p65^{Ser536} (Cell signaling, cat. no. 3033), α-GRP78 (1:500; Santa Cruz Biotechnology, cat.no. sc-13539), α-CHOP (1:1,000; Cell signaling, cat. no. 2895) α-Tubulin (1:15,000; BioCat, Germany, cat. no. G098), goat-α-mouse-HRP (1:3,000; Bio-Rad, USA, cat. no. 172-1011), goat-α-rabbit-HRP (1:3,000; Bio-Rad, USA, cat. no. 170-6515), goat-α-rat-HRP (1:5,000, Invitrogen, cat. no. 31470). For immunofluorescence the following antibodies were used: α-Actinin-2 (1:100; Abcam, UK, cat. no. ab137346), goat-α-rabbit-Alexa488 (Invitrogen, USA, cat. no. A-11008). For chromatin immunoprecipitation the following antibodies were used: α-H3K4me3 (Cell signaling, USA, cat. no. 9751), α-RNA polymerase II serine 5 phosphorylation (Abcam, UK, cat. no. ab5131), RNA polymerase II serine 2 phosphorylation (Abcam, UK, cat. no. ab5095). For immunoprecipitation the following antibodies were used: α-RyR1 (Invitrogen cat no. MA3-925), mouse isotype IgG (Invitrogen, cat no. 10400C).

Oligonucleotides

The following oligos were used for RT-qPCR:

Atp2a1 forward: 5’-AGCTTGACGAGTTTGGGGAG-3’

Atp2a1 reverse: 5’-GCGTTCTTCTTTGCCATCCG-3’

Casq2 forward: 5’-TGAGTTTGACGGGGAGTTCG-3’

Casq2 reverse: 5’-GGAAGTGTTCAGCTGCCTCT-3’

Gapdh forward: 5’-AACTTTGGCATTGTGGAAGG-3’

Gapdh reverse: 5’-ACACATTGGGGGTAGGAACA-3’

Hrc forward: 5‘-GTGAGAAGGACAGAGGCCAC-3’

Hrc reverse: 5‘-CATTGTCATGTTCCCGGTGC-3’

Myh1 forward: 5’-CAAGTCATCGGTGTTTGTGG-3’

Myh1 reverse: 5’-TGTCGTACTTGGGAGGGTTC-3’

Myh2 forward: 5’-GGCCAAAATCAAAGAGGTGA-3’

Myh2 reverse: 5’-CGTGCTTCTCCTTCTCAACC-3’

Ryr1 forward: 5‘-AGGACATGGTGGTGATGCTG-3’

Ryr1 reverse: 5‘-GCCTCAGAGCCTACGATGTC-3’

Srl isoform 1 forward: 5’-TCGGGTGGCACGGAAGATGTTG-3’

Srl isoform 1 reverse: 5’-AGGGGCCGCATCTGCATAAAGC-3’

Srl isoform 2 forward: 5’-TGCTCTGCTGTTTCCTGGCCTC-3’

Srl isoform 2 reverse: 5’-TTTGCCAACACTCCACGGTCCC-3’

Senp3 forward: 5’-AGGTGCCATCACCCTGCTG-3’

Senp3 reverse: 5’-GACTTTGGGGTCCACCTTAG-3’

Senp7 forward: 5’-AGCTTGAAGAGTCCGGTGAA-3’

Senp7 reverse: 5’-CTAGCATCTGGCTCCTGACC-3’

The following oligos were used for qPCR following chromatin immunoprecipitation:

Atp2a1 promoter forward: 5’-TTCTCAGTTCCAAGCCCACCCC-3’

Atp2a1 promoter reverse: 5’-TCTGTCCCCAAAGATGCGCTCC-3’

Myh1 promoter forward: 5’-GTGAAGGCTGCCAGAAAGAG-3’

Myh1 promoter reverse: 5’-AACACAGAGGACAGGGGATG-3’

The following oligos were used for siRNA transfection:

siSENP3: GGCCUCCUCUCAUGUACUCUA dTdT

siSENP7: CAGAGTTATGTTGACGAATGT TCTA dTdT

siGL2: CGUACGCGGAAUACUUCGA dTdT

Isolation of neonatal rat cardiomyocytes

For the isolation and cultivation of neonatal rat cardiomyocytes (NRCMs), hearts were excised out of living newborn rat pups and washed in PBS containing heparin. The hearts were minced and washed twice with PBS. Connective tissue was digested by an enzyme mix (Miltenyi Biotec, Germany, cat. no. 130-098-373) for 60 min at 37 °C on a Miltenyi gentleMACS OctoDissecter. The cells were washed several times with MEM containing 5 % FBS, centrifuged and plated on gelatin-coated coverslips. The NRCMs were incubated overnight at 37 °C and 1 % CO_2 in MEM containing 5 % FBS. The next day the cells were switched to 5 % CO_2 and DMEM containing 5 % FBS and 10 % horse serum.

Contractility assay of myotubes

Differentiated myotubes on glass coverslips were placed in a custom-made chamber containing differentiation medium set to 37 °C using a thermistor probe (Green Leaf Scientific) and a temperature controller (TC-II, micro temperature controller, Cell MicroControls). The cells were paced with the Myopacer EP field stimulator (IonOptix, USA) at 1 Hz, 40 V, and a pulse duration of 4 ms in transistor-transistor logic mode (TTL). To check the contractile propensity of the myotubes, videos of at least 15 sec duration for each view were recorded on an Olympus IX51 microscope with an optiMOS high speed camera (QImaging, Canada) attached.

Gel electrophoresis and western blotting

Whole cell lysates were collected by washing the cells with PBS and scraping in ROTI Load 1 (Carl Roth, Germany, cat. no. K929) diluted in phosphate-buffered saline (PBS; Gibco, USA, cat. no. 14190136). The samples were incubated for 7 min at 92 °C and equal amounts of protein were loaded on 10 % sodium dodecyl sulfate polyacrylamide gels (SDS-PAGE) along with Precision Plus Protein Dual Color Standard (Bio-Rad, USA, cat. no. 1610374) and separated for 70 min at 150 V in a Mini-Protean electrophoresis cell (Bio-Rad, USA, cat. no.

1658006FC). The gels were blotted on 0.45 µm nitrocellulose membranes for 60 min at 10 V in a semi-dry blotter (Bio-Rad, USA, cat. no. 1703940). For RyR1-blots, whole cell lysates were loaded on 7.5 % SDS-PAGEs and separated for 90 min at 150 V along HiMark pre-stained protein standard (Invitrogen, USA, cat. no. LC5699). Proteins were transferred on activated 0.45 µm polyvinylidene difluoride membranes (Thermo Scientific, USA, cat. no. 88518) in a Criterion wet blotting system (Bio-Rad, USA, cat. no. 1704070) overnight at 4 °C and 20 V. After blocking for 60 min at room temperature in 4 % (w/v) non-fat dry milk (ChemCruz, USA, cat. no. SC-2325) in TBST (150 mM NaCl (Supelco, USA, cat. no. 1.06404), 50 mM Tris (Sigma-Aldrich, USA, cat. no. T1503), pH 7.4, 0.1 % (v/v) Tween-20 (Sigma-Aldrich, USA, cat. no. P1379)) primary antibodies were incubated overnight at 4 °C in blocking solution with gentle agitation. After washing in TBST, secondary antibodies were incubated for 60 min at room temperature in blocking solution. The blots were developed with SuperSignal West Dura (Thermo Scientific, USA, cat. no. 34075) and imaged on an ImageQuant LAS4000 System.

RNA-sequencing

500 ng RNA per sample was enriched using 'NEBNext® Poly(A) mRNA Magnetic Isolation Module' (E7490L; New England Biolabs) followed by stranded cDNA library generation using 'NEBNext® Ultra II Directional RNA Library Prep Kit for Illumina' (E7760L; New England Biolabs) according to the manualE7760 (Version 1.0_02-2017; NEB), except that reactions were reduced to 2/3 of initial volumes. cDNA libraries were barcoded using 'NEBNext Multiplex Oligos for Illumina – 96 Unique Dual Index Primer Pairs' (6440S; New England Biolabs), cDNA libraries were amplified with 7 cycles of final pcr and additionally purified using 1.2x 'Agencourt® AMPure® XP Beads' (#A63881; Beckman Coulter, Inc.). 'Bioanalyzer High Sensitivity DNA Assay' (5067-4626; Agilent Technologies) was used for fragment length distribution and all libraries were quantified with the 'Qubit® dsDNA HS Assay Kit' (Q32854; ThermoFisher Scientific). Equal molar amounts of all libraries were pooled for a common sequencing run, denatured with NaOH and was diluted to 1.8 pM according to the Denature and Dilute Libraries Guide (Document # 15048776 v02; Illumina). 1.3 ml of the denatured pool

was loaded on an Illumina NextSeq 550 sequencer using a High Output Flowcell (400M cluster) for single reads (20024906; Illumina). Sequencing was performed with the following settings: Sequence reads 1 and 2 with 38 bases each; Index reads 1 and 2 with 8 bases each. BCL files were converted to FASTQ files using bcl2fastq Conversion Software version v2.20.0.422 (Illumina). Raw data was processed with nfcore/rnaseq (version 1.4.2), using Nextflow. The genome reference and annotation data were taken from GENCODE.org (*Mus musculus*; GRCm38.p6). Normalization and differential expression analysis was performed on the internal Galaxy (version 20.05) instance of the RCU Genomics, Hannover Medical School, Germany using DESeq2 (Galaxy Tool Version 2.11.40.6) at default settings with the following exceptions: “Output normalized counts table” was set to “Yes” and additional filters were disabled (“Turn off outliers replacement”, “Turn off outliers filtering”, and “Turn off independent filtering” set to “Yes”). Cut-offs for identification of differentially expressed genes (DEGs) were set to $\log_2$ fold change of 0.7 and an adjusted p-value of ≤ 0.05. Ontology enrichment analysis was performed with the Database for Annotations, Visualization and Integrated Discovery (DAVID)[S1] and metascape [S2]. Selected ontology terms were visualized in a circle and chord plot with the GOplot package for R[S3].

Immunofluorescence assay and confocal imaging

Differentiated myotubes on glass coverslips were fixated at room temperature for 15 min in 4 % (w/v) paraformaldehyde in PBS (Thermo Scientific Chemicals, USA, cat. no. J19943) and washed twice in PBS. The cells were permeabilized for 15 min in 0.2 % (v/v) Triton X-100 (Roche, Switzerland, cat. no. 11332481001) and blocked for 30 min in 5 % (w/v) bovine serum albumin (Gerbu Biotechnik, Germany, cat. no. 1063) in PBS. Primary antibodies were incubated at room temperature for 60 min in blocking solution, washed twice with PBS, followed by incubation with secondary antibodies as above. Nuclei were stained for 5 min with 4’-6-Diamidino-2-phenylindole (DAPI; Sigma, USA, cat. no. D9542). The coverslips were mounted on glass slides with Fluoroshield (Sigma, USA, cat. no. F6182). Images were collected on an Olympus FV1000 confocal microscope. The primary Myotubes cultured on 8-

well chamber slide were fixed using 4 % PFA for 10 min at room temperature (RT), followed by three washes of 3 min each in PBS 1X and permeabilization using 0.5% (v/v) Triton X-100 for 10 min at RT. Myotubes were then washed twice in PBS 1X and non-specific signals were blocked with 1% BSA + 2.5% of goat serum in PBS 1X for 1 h at RT. The primary antibody against sarcomeric alpha-actinin (Abcam, ab137346) was diluted 1:100 in blocking solution and incubated 1 h at RT. Myotubes were incubated with the secondary antibody (Rb568) diluted 1:500 in blocking solution for 1 h at RT in dark condition. After washing, the myotubes were incubated with DAPI for 10 minutes at RT in the dark. Images were acquired using the 40X and 60X oil immersion objective of the Confocal Leica SP5 at the Imaging Facility in INGM, Italy.

Reverse transcription quantitative polymerase chain reaction (RT-qPCR)

C2C12 myotubes from 6-well plates were collected with TrypLE (Gibco, USA, cat. no. 12604013) and PBS and RNA was extracted with the High Pure RNA Isolation Kit (Roche, Switzerland, cat. no. 11828665001) according to the manual. cDNA was synthesized using the Maxima First Strand cDNA Synthesis Kit (Thermo Scientific, USA, cat. no. K1641) according to the manual. Quantitative polymerase chain reaction (qPCR) was performed using the PowerUp SYBR Green Master Mix (Applied Biosciences, USA, cat. no. A25741) on a QuantStudio 6 Flex System (Thermo Fischer, USA). The cycling program was 95 °C for 8 min, 45 cycles of [95 °C for 15 sec, 60 °C for 60 sec]. mRNA expression was quantified using the $\Delta\Delta C_T$ method and normalized to *Gapdh* gene expression.

Immunoprecipitation

Immunoprecipitation was performed as in ref [S4]. In short, myotubes were collected and lysed for 25 min at 4 °C in 50 mM HEPES (pH 7.5), 150 mM NaCl, 1 mM EDTA, 1 mM EGTA, and 0.5 % (v/v) Triton X-100 in presence of protease and phosphatase inhibitor. The lysates were centrifuged for 15 min at 16,000 g and 4 °C and the supernatants incubated overnight at 4 °C with 8 µg RyR1 antibody (Invitrogen cat no. MA3-925) or mouse isotype IgG (Invitrogen, cat no. 10400C). Antibody complexes were collected with protein G dynabeads for 60 min 4 °C

and washed four times on a magnetic rack with 1/4x lysis buffer. Samples were eluted by incubation for 7 min at 92 °C with SDS-PAGE sample buffer.

siRNA transfection and plasmid transfection

Transfection of siRNA against SENP3 and SENP7 was performed as previously described [S4,5]. In short, C2C12 cells were transfected on day 2 of differentiation with 100 pmol of the indicated siRNA using Lipofectamine LTX according to the manual. The cells were collected on day 5 of differentiation. For plasmid transfection (1 µg of either pcDNA3.1-SENP3; pcDNA3.1-SENP3 or empty vector control) we transfected cells by using via Lipofectamine® LTX & PLUS™ Reagent Protocol (Catalog number A12621, Thermofisher Scientific).

References

S1. Sherman BT, Hao M, Qiu J, Jiao X, Baseler MW, Lane HC *et al.* DAVID: a web server for functional enrichment analysis and functional annotation of gene lists (2021 update). *Nucleic Acids Res* 2022;**50**:W216–W221.

S2. Zhou Y, Zhou B, Pache L, Chang M, Khodabakhshi AH, Tanaseichuk O *et al.* Metascape provides a biologist-oriented resource for the analysis of systems-level datasets. *Nat Commun* 2019;**10**.

S3. Walter W, Sánchez-Cabo F, Ricote M. GOplot: An R package for visually combining expression data with functional analysis. *Bioinformatics* 2015;**31**:2912–2914.

S4. Amrute-Nayak M, Gand LV, Khan B, Holler T, Kefalakes E, Kosanke M *et al.* SENP7 deSUMOylase-governed transcriptional program coordinates sarcomere assembly and is targeted in muscle atrophy. *Cell Rep* 2022;**41**:111702.

S5. Nayak A, Lopez-Davila AJ, Kefalakes E, Holler T, Kraft T, Amrute-Nayak M. Regulation of SETD7 Methyltransferase by SENP3 Is Crucial for Sarcomere Organization and Cachexia. *Cell Rep* 2019;**27**:2725-2736.e4.
